# Kinetics of cardiac troponin and other biomarkers in patients with ST elevation myocardial infarction

**DOI:** 10.1016/j.ijcha.2023.101250

**Published:** 2023-08-09

**Authors:** Jonas Henrik Kristensen, Clara Amalie Wistisen Koczulab, Emil Anton Frandsen, Rasmus Bo Hasselbalch, Nina Strandkjær, Nicoline Jørgensen, Morten Østergaard, Peter Hasse Møller-Sørensen, Jens Christian Nilsson, Shoaib Afzal, Pia Rørbæk Kamstrup, Morten Dahl, Mustafa Vakur Bor, Ruth Frikke-Schmidt, Niklas Rye Jørgensen, Line Rode, Lene Holmvang, Jesper Kjærgaard, Lia Evi Bang, Julie Forman, Kim Dalhoff, Henning Bundgaard, Kasper Karmark Iversen

**Affiliations:** a Department of Cardiology, Copenhagen University Hospital - Herlev and Gentofte, Borgmester Ib Juuls vej 1, 2730 Herlev, Denmark; b Department of Emergency Medicine, Copenhagen University Hospital - Herlev and Gentofte, Borgmester Ib Juuls vej 1, 2730 Herlev, Denmark; c Department of Clinical Medicine, University of Copenhagen, Blegdamsvej 3B, 2200 Copenhagen, Denmark; d Department of Cardiology, Copenhagen University Hospital - Rigshospitalet, Blegdamsvej 9, 2100 Copenhagen, Denmark; e Department of Clinical Pharmacology, Copenhagen University Hospital – Bispebjerg and Frederiksberg, Bispebjerg Bakke 23, 2400 Copenhagen, Denmark; f Department of Cardiothoracic Anaesthesiology, Copenhagen University Hospital - Rigshospitalet, Blegdamsvej 9, 2100 Copenhagen, Denmark; g Department of Clinical Biochemistry, Copenhagen University Hospital - Herlev and Gentofte, Borgmester Ib Juuls vej 1, 2730 Herlev, Denmark; h Department of Clinical Biochemistry, Zealand University Hospital – Køge, Lykkebækvej 1, 4600 Køge, Denmark; i Department of Clinical Biochemistry, University Hospital of Southern Denmark, Finsensgade 35, 6700 Esbjerg, Denmark; j Department of Clinical Biochemistry, Copenhagen University Hospital – Rigshospitalet, Blegdamsvej 9, 2100 Copenhagen, Denmark; k Section of Biostatistics, Department of Public Health, University of Copenhagen, Øster Farimagsgade 5, 1353 Copenhagen, Denmark

**Keywords:** Cardiac troponin, Cardiac biomarkers, Kinetics, Myocardial infarction, Coronary artery disease, Cohort study

## Abstract

**Objective:**

To examine changes in concentration, time-to-peak and the ensuing half-life of cardiac biomarkers in patients with myocardial infarction.

**Methods:**

Blood sampling was performed every third hour within 24 h after percutaneous coronary intervention (PCI) on a cohort of patients with ST elevation myocardial infarction. Cardiac troponin (cTn) was measured by the Dimension Vista, Vitros, Atellica, and Alinity high-sensitivity (hs) cTnI assays, and the Elecsys hs-cTnT assay. Further, creatine kinase (CK), myoglobin, creatine kinase MB (CKMB) and other biomarkers were analyzed.

**Results:**

A total of 36 patients completed blood sampling (median age 60 years, IQR 56.4–66.5 years; seven women, 19.4%). Hs-cTnI measured by the Vitros assay was the first hs-cTn to peak at 9.1 h (95%-CI 6.2–10.1) after PCI and 11.7 h (95%-CI 10.4–14.8) after symptoms onset. There were no notable differences between hs-cTn assays in regard to time-to-peak. Also, Vitros hs-cTnI reached the highest median ratio of concentration to upper reference level of nearly 2,000. The median half-life from peak concentration ranged from 7.6 h for myoglobin (CI 6.8–8.6) to 17.8 h for CK (CI 6.8–8.6). For hs-cTn assays the median T½ ranged from 12.4 h for the Vista hs-cTnI assay (95%-CI 11.0–14.1 h) to 17.3 h for the Elecsys hs-cTnT (95%-CI 14.9–20.8 h).

**Conclusions:**

This study updates knowledge on the kinetics of cardiac biomarkers in current clinical use. There was no notable difference in trajectories, time-to-peak or half-life between hs-cTn assays.

## 1 Introduction

Biomarkers of myocardial injury are crucial in the evaluation of patients with chest pain [1]. Cardiac biomarkers represent a cornerstone in the diagnosis of myocardial infarction but have also proven to be valuable when diagnosing other cardiac conditions such as myocarditis [2].

The kinetics of cardiac biomarkers have previously been examined, especially the older assays for cardiac troponin (cTn) as well as creatine kinase MB (CKMB) [3]. Both cTnI and cTnT concentrations have been shown to peak at a median of 11–13 h after hospital admission but the timing has a high degree of interindividual variance [1], [4], [5]. Nevertheless, the dynamic changes in cTnI and cTnT in the blood stream following myocardial infarction have been found to differ in the later stage due to a secondary peak of cTnT at a median of 77–82 h after hospital admission [4], [5]. Dynamic changes in cTn over time are used to distinguish acute myocardial injury from chronically elevated cTn. However, a comparison of the kinetics between different cardiac biomarkers in current clinical use is warranted as the dynamic changes are important to correctly diagnose patients with chest pain [6].

Clearance of cTn from the extracellular space is thought to be mostly extrarenal, especially at high concentrations [7]. Extrarenal clearance of proteins is often driven by scavenger receptor-mediated endocytosis by phagocytes [8]. However, at lower concentrations it seems that renal excretion plays a role as suggested by a study in rats [7]. Another study found cTn in the urine of patients with myocardial infarction without renal disease confirming the assumption that renal excretion occurs [9]. Due to the prolonged release of cTn during myocardial infarction the metabolization and half-life (T½) of cTn in plasma is still largely unknown.

The purpose of this study was to update knowledge on kinetics of cardiac biomarkers in patients with myocardial infarction. We compared changes in concentration over time, time-to-peak and the ensuing rate-of-decay of five different high-sensitivity (hs) cTn assays as well as creatine kinase (CK), myoglobin, CKMB, lactate dehydrogenase (LDH), C-reactive protein (CRP) and alanine transaminase (ALT).

## 2 Methods

In this prosepctive cohort study we performed repeated blood sampling on patients with ST elevation myocardial infarction (STEMI) at the Department of Cardiology, Rigshospitalet, Denmark.

### 2.1 Patients

Admitted patients were screened and asked to participate after acute percutaneous coronary intervention (PCI) and inclusion criteria were: 1) STEMI in patients ≥ 18 years of age, 2) inclusion < 24 h after PCI 3) ability to give informed consent, 4) no renal failure defined as estimated glomerular filtration rate (eGFR) < 15 ml/min/1.73 m^2^ or receiving dialysis, 5) hemodynamic stability (mean arterial pressure > 70 mmHg and no use of inopressors), 6) one or more luminal coronary artery stenoses of ≥ 70%. Only patients with a hs-cTnT concentration > 500 ng/L upon admission or > 1,000 ng/L within the last 12 h were included.

### 2.2 Patient involvement

Patients were not involved in formulating the research questions or how to disseminate the results, but they were involved in design and implementation of the blood sampling schedule as well as recruitment to the study.

### 2.3 Blood sampling

Remaining blood from clinic sampling was collected from PCI to the time of consent. Study blood sampling continued at fixed intervals of three hours until 24 h after PCI. Samples were predominantly collected from a peripheral venous catheter. If this was not possible a new peripheral venous catheter was inserted, or sampling was performed with a regular blood collection kit. Vials containing lithium-heparin were used.

All blood samples were kept in a refrigerator until centrifugation. They were centrifuged 30 min to eight hours after extraction. Centrifugation was performed at a relative centrifugal force of 1,520 × g for 10 min at room temperature. Aliquots of plasma were then stored at −80° C. The following methods were used for biochemical analyses [10].

### 2.4 Biochemical analysis

The Alinity i STAT hs-cTnI assay (Abbott Laboratories, Chicago, the United States of America) is an automated, two-step immunoassay for the quantitative determination of hs-cTnI in human plasma using chemiluminescent microparticle immunoassay technology. The hs-cTnI assay was measured on the Alinity analyzer.

The Dimension Vista hs-cTnI assay (Siemens, München, Germany) is a one-step sandwich chemiluminescent immunoassay using three sheep/mouse monoclonal antibodies: one capture antibody specific for the amino acid chain epitopes 29–34 and two detection antibodies specific for the amino acid chain epitopes 41–50 and 171–190, respectively. The hs-cTnI assay was measured on Dimension Vista 1500 analyzer.

The Vitros hs-cTnI assay (Ortho Clinical Diagnostics, New Jersey, the United States of America) is an immunometric assay using three mouse monoclonal antibodies: one capture antibody specific for the amino acid chain epitopes 87–90 and two detection antibodies specific for the amino acid chain epitopes 24–40 and 41–49, respectively. The hs-cTnI assay was measured on Vitros 5600 analyzer.

The Atellica hs-cTnI assay (Siemens, München, Germany) is an immunoassay using two capture antibodies (mouse and sheep), specific for the amino acid chain epitopes 41–50 and 171–190, and one detection antibody specific for the amino acid chain epitopes 29–34. The hs-cTnI assay was measured on Atellica IM 1600 Analyzer.

Hs-cTnT was measured using the Elecsys immunoassay (Roche Diagnostics, Basel, Germany) and a Cobas 8000 analyzer system according to the manufacturer’s instructions.

Lactate dehydrogenase (LDH), alanine transferase (ALT), and CK were measured using enzymatic assays with photometric detection (Atellica CH Analyzer). Hs C-reactive protein (CRP) was measured using an immunotubidimetric assay (Atellica CH Analyzer). Myoglobin was measured using turbidimetry and CKMB was measured using a sandwich electrochemiluminescens immunoassay with photometric detection, both on a Cobas 8000 analyzer.

Some concentrations of hs-cTnI measured on the Atellica and Vista hs-cTnI assays were reported as > 125.000 ng/l and due to a lack of plasma for dilution these could not be determined further. The same applied to two analyses of CK-MB which were > 600 µg/l. These concentrations were set to the stated value. All other measurements which could not be biochemically determined were censored. All upper reference levels (URL) followed the values implemented in the clinic (see supplementary appendix p. 2 for further information).

### 2.5 Ethics

This study was registered with the Danish Data Protection Authorities (VD-2019–172). The study protocol conforms to the ethical guidelines of the Declaration of Helsinki and was approved by the Regional Scientific Ethics Committee of the Capital Region (H-19065459). Written informed consent was obtained from all participants.

### 2.6 Data management and analysis

Data was managed using Research Electronic Data Capture (REDCap), a secure, web-based, electronic data capture tool, hosted at the Capital Region’s server [11], [12]. Descriptive statistics are reported as median with interquartile range (IQR) for quantitative data and number with percentage for categorical data. All biomarker concentrations were log-transformed prior to analysis due to substantial skewness. Median concentrations over time with 95% confidence intervals (CI) were estimated using a linear mixed model with a fixed effect of time and an unstructured covariance pattern. Missing data was implicitly handled by maximum likelihood estimation in the linear mixed model. To examine differences of the kinetic profiles of the hs-cTns, CKMB, CK and myoglobin, concentrations were plotted as percentages of the maximum concentration as estimated by linear mixed model.

Time-to-peak was computed for each study participant in turn and summarized as median with 95% CI. A post exploratory paired *t*-test of time to peak from PCI for hs-cTnT vs all hs-cTnI measurements was calculated and reported with 95%-CI. As an acute phase response has been shown to impact the Vitros hs-cTnI assay, we performed a post exploratory sensitivity analysis of time to peak stratifying participants according to CRP <=20 mg/L versus those with CRP > 20 mg/L for all hs-cTn assays [13].

Median rate-of-decay and T½ after peak with 95% CI was estimated using a linear mixed model. Comparisons between assays were made using forest plots. Model assumptions including log-linear decay after peak time were assessed using residual diagnostics. Data analysis was done in R (version 4.2.2). The asht-package (version 1.0.0) was used to compute exact 95% CIs for medians [14].

## 3 Results

**Fig. 1:**
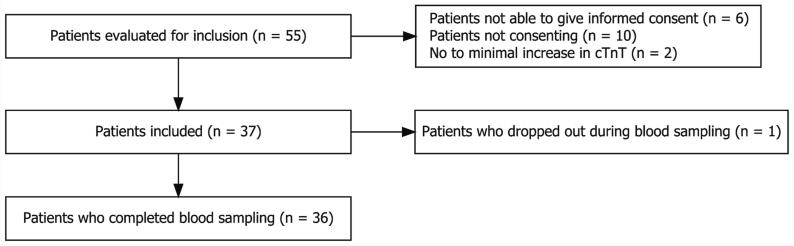
Flow chart of study inclusion and completion.

From February 10, 2021, to April 8, 2021, a total of 55 patients were screened for study eligibility after PCI. Six patients were not able to provide consent and ten patients did not want to participate. Two patients did not reach the minimal hs-cTnT concentration. One patient dropped out after nine hours of sampling due to exhaustion, and hence was excluded from analysis because of insufficient data. Thus, 36 participants completed the study (Fig. 1).

Median age was 59.63 years (IQR 56.4–66.46 years) and seven were women (19.4%) (Table 1). Chronic heart disease (n = 12, 33.3%) and essential hypertension (n = 20, 55.6%) were the most frequent comorbidities. The median time from onset of symptoms to start of PCI was 2.38 h (IQR 1.93–4.36). Median left ventricular ejection fraction was 42.5% (IQR 35–50) and 27 had a luminal stenosis of at least one coronary artery of ≥ 99% (75%). A secondary angiography was performed in four patients to evaluate suitability for coronary artery bypass grafting or inclusion in other clinical studies. Of these four, one patient had a drug-eluting stent implanted at a secondary PCI (supplementary figure S1). No patients became hemodynamic unstable or experienced cardiac arrest during the study.

**Table 1:** Baseline characteristics.

	Overall
**n**	36
**Women (%)**	7 (19.4)
**Age in years (median [IQR])**	59.63 [56.40, 66.46]
**Body mass index (median [IQR])**	27.07 [25.54, 32.61]
**Ever smoker (%)**	28 (77.8)
**Excess alcohol consumption^(%)**	5 (13.9)
**Ejection fraction as percentage (median [IQR])**	42.50 [35.00, 50.00]
**Coronary artery luminal stenosis ≥ 99% (%)**	27 (75.0)
**European ancestry (%)**	34 (94.4)
**Time from symptoms onset to PCI in hours (median [IQR])**	2.38 [1.93, 4.36]
**Chronic lung disease (%)**	4 (11.1)
**Diabetes mellitus (%)**	6 (16.7)
**Chronic heart disease (%)**	12 (33.3)
**Chronic kidney disease (%)**	1 (2.8)
**Impaired immune system (%)**	2 (5.6)
**Hypercholesterolemia (%)**	9 (25.0)
**Hypertension (%)**	20 (55.6)
**Medication at admission**	
**Antihypertensive medication (%)**	17 (47.2)
**Statin or fibrate medication (%)**	12 (33.3)
**Acetylsalicylic acid (%)**	8 (22.2)
**ADP-receptor inhibitors (%)**	2 (5.6)
**Diuretic medication (%)**	2 (5.6)
**Antidiabetic medication (%)**	5 (13.9)
**Thyroid substitution medication (%)**	1 (2.8)
**Antidepressant medication (%)**	4 (11.1)
**Anxiolytic or anti-epileptic medication (%)**	4 (11.1)
**Astma medication (%)**	3 (8.3)
**Immunosuppressant medication (%)**	2 (5.6)
**Chemotherapy (%)**	1 (2.8)
**Education (%)**	
**No education, elementary school or high school**	13 (36.1)
**Vocational education or bachelor’s degree**	19 (52.8)
**Master’s degree or higher**	4 (11.1)

Baseline characteristics. PCI: primary percutaneous coronary artery intervention.

^Excess alcohol consumption was defined as>10 units of alcohol per week according to the current Danish guidelines [32].

### 3.1 Trajectories of cardiac biomarkers over time

**Fig. 2:**
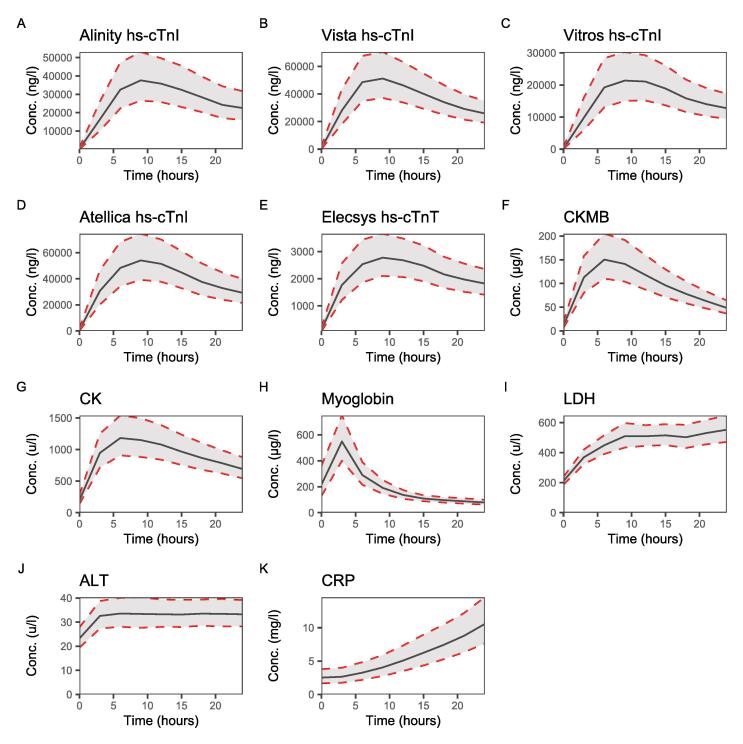
Median concentration of cardiac biomarkers over time.

Within the first 24 h after PCI a notable peak due to a rise and fall was observed for all hs-cTn assays, CKMB, CK, and myoglobin (Fig. 2). A rise of LDH was also observed while a slight rise was observed for CRP and ALT. Spaghetti plots of individual participant concentrations are appended in the supplementary material (supplementary figure S2-12). All hs-cTn assays followed a similar trajectory during the first 24 h (supplementary figure S13 and S14). Myoglobin was the first biomarker to peak 3.10 h after PCI (95%-CI 2.93–3.43).

### 3.2 Magnitude of elevation of cardiac biomarkers

Ratios of concentrations divided by the URLs were plotted for all cardiac biomarkers to examine the elevations between biomarkers (supplementary figure S15).

Hs-cTn assays reached median concentrations>100 times above the URL while the Vitros hs-cTnI assay reached the highest ratio of concentration/URL (nearly 2,000) (supplementary figure S16).

. CK, CKMB and myoglobin reached median concentrations 5–20 times the URL, LDH and CRP reached median concentrations two to three times the URL, and median concentration of ALT did not reach the URL.

### 3.3 Time to peak of cardiac biomarkers

**Fig. 3:**
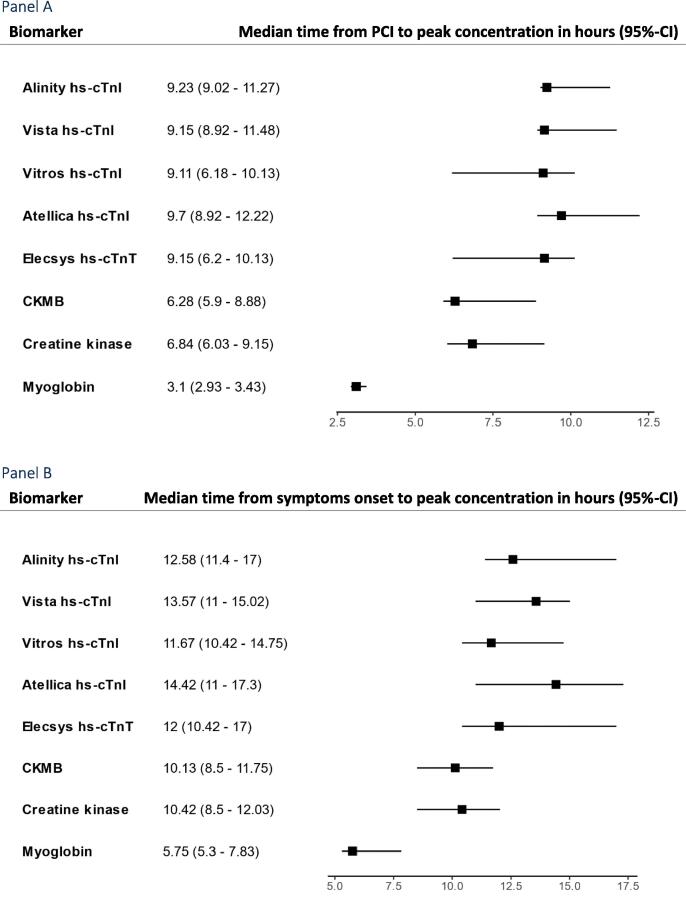

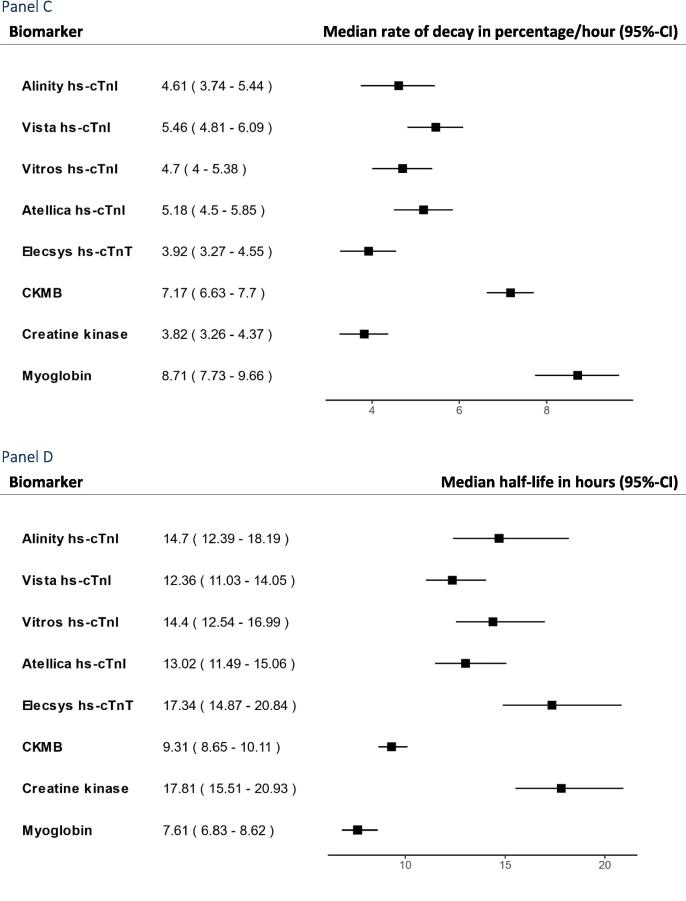
Forrest plots of time to peak, rate of decay and half-life for high sensitivity cardiac troponins, creatine kinase, CKMB, and myoglobin.

The first hs-cTn to peak was hs-cTnI measured by the Vitros hs-cTnI assay which peaked at 9.11 h (95%-CI 6.18–10.13) after PCI and 11.67 h (95%-CI 10.42–14.75) after symptoms onset (Fig. 3). The last hs-cTn assay to peak was the Atellica hs-cTnI assay which peaked at 9.7 h (95%-CI 8.92–12.22) after PCI and 14.42 h (95%-CI 11–17.3) after symptoms onset. There were no notable differences between hs-cTn assays. Myoglobin peaked first at a median of 3.1 h (95%-CI 2.93–3.43) after PCI and 5.75 h (95%-CI 5.3–7.83) after symptoms onset. The peak of myoglobin after PCI was about six hours before any hs-cTn assay. A sensitivity analysis stratifying participants according to CRP > 20 (n = 7) or CRP=<20 (n = 29) did not find a significantly shorter time to peak for participants with CRP > 20 (supplementary appendix figure S19). When examining the difference between cTnT and all cTnI measurements, cTnT peaked 0.65 h earlier (95%-CI −1.61–0.3).

The bar plot in supplementary figure S17 shows when study participants reached their peak concentration after PCI for each biomarker peaking in the observation period. Again, myoglobin stood out as > 75% of participants reached the peak myoglobin concentration after three hours while nine to 12 h passed before participants reached their peak concentration for the five hs-cTn assays.

### 3.4 Decay of cardiac biomarkers in the clinic

Supplementary figure S18 shows decay of all hs-cTn assays, myoglobin, CK and CKMB in the bloodstream. As LDH, ALT and CRP did not show a peak including a rise and fall within the first 24 h, these biomarkers are not presented. Decay was set to start at the individual participant peak concentration and available measurements were used up until 15 h after. Participants with only one measurement were censored resulting in available data for 33–36 participants for the various biomarkers. The median T½ from peak of concentration ranged from 7.6 h for myoglobin (95%-CI 6.8–8.6) to 17.8 h for CK (95%-CI 15.5–20.1). For hs-cTn assays the median T½ ranged from 12.4 h for the Vista hs-cTnI assay (95%-CI 11.0–14.1 h) to 17.3 h for the Elecsys hs-cTnT (95%-CI 14.9–20.8 h) (Fig. 3, panel D).

A sensitivity analysis of time to peak, rate of decay and half-life was performed excluding participants with eGFR < 90 ml/min/1,73 m^2^ leaving 22 participants with an eGFR of > 89 ml/min/1,73 m^2^ (appendix, figure S20).

## 4 Discussion

The main findings in this study were 1) in the five applied hs-cTn assays concentrations followed a similar pattern of changes during the first 24 h after PCI; 2) median time from PCI to max hs-cTn concentration was nine to ten hours while median time from symptoms onset to max concentration of hs-cTn was 12 to 14 h for all hs-cTn assays; 3) there was no notable difference in time-to-peak between hs-cTn assays; 4) elevations relative to the URL were highest for hs-cTn assays and the highest elevation was found for the Vitros hs-cTnI assay; 5) decay of hs-cTn followed similar trajectories for all hs-cTn assays; 6) the T½ ranged between eight hours for myoglobin to 18 h for CK; 7) for hs-cTn assays exclusively, the T½ ranged from 12 h for the Vista hs-cTnI assay to 17 h for the Elecsys hs-cTnT assay.

The results of this study may help guiding doctors when evaluating patients with STEMI. The half-life of cTn is useful when evaluating ongoing myocardial injury and when the release of cTn from cardiomycytes has stopped. Great variability of the course of cTn is, however, expected between individuals but variability of the course between hs-cTn assays was low. This may be helpful when patients are transferred between centers which use different assays although standardized scaling of cTn does not exist for hs-cTn assays. The findings indicate that hs-cTnI and hs-cTnT follow the same kinetics which may be used in some instances to generalize research on one hs-cTn assay to another.

The kinetics of all hs-cTn assays followed similar trajectories which strengthens the assumption that the release of cTnI and cTnT, and the following degradation in the blood stream, is very much alike in the aftermath of myocardial infarction. Studies using *in vitro* models of release of cTnI and cTnT from cardiomyocytes subjected to ischemia have found the release to begin during cell necrosis three to six hours after induced ischemia [3], [15], [16]. In these studies, cTn was not released from viable cells. However, in a previous clinical study, elevated levels of cTnI and cTnT were detectable already at 15 min after induction of myocardial ischemia [17]. It is a continued debate whether the early release is due to reversible or irreversible myocardial injury.

CTn has been hypothesized to be released due to other processes than myocardial infarction such as cardiomyocyte renewal, increased membrane permeability, and attachment of intracellular vesiculas to the cell membrane leading to ejection of vesicular molecules into the blood stream [6]. Several physiological pathways have been found to explain an elevation of the concentration of cTn without cardiomyocyte necrosis. Stretch of cardiomyocytes has been demonstrated to be associated with release of cTn to the bloodstream clinically, and an *in vitro* cell model found integrins to be mediators without any cell necrosis [18], [19]. cTnT has also been shown to be expressed in skeletal muscle tissue in patients with chronic kidney disease which may be related to elevation of the concentration in the bloodstream of these patients [20]. These studies highly suggest that the pathway to release of cTn from cardiomyocytes differs for different underlying pathophysiologies. This may influence the kinetics of cTn release, the T½ in the blood stream, and whether cTn has already partly been degraded intracellularly. Therefore, our results apply solely to the release and following decay in the bloodstream in relation to myocardial infarction [6].

The release of cTn may further vary due to individual variation. Starnberg et al. demonstrated in an *in vitro* study that release of cTnT may be accelerated when localized blood flow is high or distribution volume is large [21].

We found a notably shorter time to max concentration for myoglobin compared to all hs-cTn assays. Myoglobin has previously been found to be an early predictor of myocardial injury [22]. De Winther et al. showed a negative predictive value of myoglobin of 89% four hours after the onset of symptoms [22]. The fast rise and decline of myoglobin compared to cTn may be due to differences in molecular weight (17 vs 29–37 kDa) [23], [24], solubility, and binding of cTn to the myofibrils [21]. However, all patients were subjected to PCI, and the fast rise and fall of myoglobin concentration could be caused by the performed intervention. Also, myoglobin and CK may be released from skeletal muscle. Due to the strict inclusion criteria where only participants with high cTnT concentrations, visualized stenosis on CAG and ST elevation on ECG, the rise of myoglobin and CK in this study was assumed to be caused by myocardial injury instead of skeletal muscle damage. Possible simultaneous skeletal muscle damage can however not be completely ruled out.

The T½, as determined by this study, is clinically relevant, but it does not represent the true T½ of cTnI and cTnT in humans due to ongoing leakage of cTn from the myocardium which makes it impossible to calculate the true T½. When there are two unknown variables, i.e. influx of cTn from the myocardium and rate of decay in the blood stream measured by the T½, one cannot estimate either.

This may be an explanation of the huge discordance between estimated T½ in animal models and what can be observed clinically in patients with myocardial infarction. A previous study found a T½ of cTnI in patients with myocardial infarction (n = 22) of 20.4 (SD 10.7) and 6.8 (SD 5.6) hours according to if Q-waves were present or not, respectively [25]. Studies using animal models where exogenous cTnI is injected have shown a much shorter T½ in dogs (1.9 h), rats (0.8 h) and horses (0.5 h) [26], [27]. The use of exogenous, recombinant cTn may, however, lead to immune-mediated degradation which could shorten the T½. Thus, both species-specific differences as well as the use of exogenous cTn challenges the extrapolation to humans.

In this study, the start of PCI was used as starting point when determining timing of sampling. Symptoms onset may be a more biologically sound starting point but may also be prone to individual patient interpretation due to stuttering or misregistration of symptoms.

The concentrations of ALT and LDH are known to increase during myocardial infarction [28], and although these markers are not recommended in the work-up of patients with myocardial infarction [29], we included them to examine the kinetics of these biomarkers during STEMI. It is important for clinicians to know how other biomarkers act in the presence of STEMI. Especially as these biomarkers are often performed routinely during hospital admittance.

### 4.1 Strengths and limitations

This study was strengthened by strict inclusion criteria to increase participant comparability as well as frequent sampling during the first 24 h after PCI to get a detailed view of biomarker kinetics. The simultaneous sampling of all biomarkers allowed direct, paired comparison of these. Also, samples were drawn during nighttime to account for diurnal changes in hs-cTnT as previously reported [30], [31]. Nighttime sampling was uncommon in previous studies of the concentration of cTn over time [4], [5]. This study was limited by a moderate population size which we, however, found suitable for the main purpose of describing the kinetics. Included patients were diagnosed with STEMI and the findings may not be transferable to patients with NSTEMI.

We used time to peak to describe the kinetic profile of cardiac biomarkers, which is independent of differences across sex, age and populations instead of time to reaching the URL. As patients may have already passed the URL before sampling started and myocardial injury is not only defined by the URL but also the dynamic changes, we found time to peak to be more suitable for the purpose of this study.

The high cTnT cutoffs were employed to ensure that participants experienced ischemia resulting in myocardial necrosis. The kinetics of cTn may be affected by the process mediating release of cTn from the myocardium and in this study, we wanted to examine the kinetics following STEMI [6]. The high cutoff for cTn was thus employed to exclude cases without necrosis due to very short duration of ischemia. Ischemia without necrosis can mediate release of troponin [17], which may be due to e.g. exertion, and not an occlusion of the coronary arteries. Also, the decay of cTn may be affected by the concentration of cTn [7]. We therefore wanted participants to reach a high concentration of cTn.

## 5 Conclusions

In this clinical study on the kinetics of cardiac biomarkers we described the changes of concentrations of cardiac biomarkers during the first 24 h acute PCI. Further research is needed to determine the true T½ of cTnI and cTnT in the blood stream.

## Declaration of Competing Interest

The authors declare the following financial interests/personal relationships which may be considered as potential competing interests: [HB received payment or honoraria for lectures, presentations, speakers bureaus, manuscript writing or educational events from Amgen, Sanofi, BMS, MSD. HB owns stock or stock options in Novo Nordic. PRK reported a grant from Gangsted Fonden outside the present work. PRK reported consulting fees from Novartis and Silence Therapeutics. PRK reported Payment or honoraria for lectures, presentations, speakers bureaus, manuscript writing or educational events from Physicians’ Academy for Cardiovascular Education, Novartis and PCSK9 Forum. RFS reported grants or contracts outside the present work from Lundbeck Foundation, Innovation Fund Denmark, The Danish Heart Foundation, Sygeforsikringen Denmark Research Fund, Leducq Foundation. RFS reported payment or honoraria for lectures, presentations, speakers bureaus, manuscript writing or educational events from Novo Nordic. RFS reported being a steering committee member of the Copenhagen General Population Study, Steering committee member of the Copenhagen Baby Heart Study, and Deputy Head, Department of Clinical Medicine, University of Copenhagen. JK reports a grant or contract from Novo Nordic outside the present work. LH reported personal payment or honoraria for lectures from Boeringer Ingelheim and payment or honoraria to her institution for lectures from Bayer. LH reported receiving support for travel from Abbott to her Institution. MVB reported receiving honoraria for a lecture from Bristol-Myers.].
