# Supplementary material for: Kinetics of cardiac troponin and other biomarkers in patients with ST elevation myocardial infarction
Source: Int J Cardiol Heart Vasc. 2023 Aug 9;48:101250. doi: 10.1016/j.ijcha.2023.101250 (PMC10432699; doi:10.1016/j.ijcha.2023.101250)
Supplement: Supplementary data 1 [file mmc1.docx]

Supplementary Appendix

Table of contents

[Information on upper reference levels and stability of biomarkers 2](#_Toc137822509)

[Figure S1 individual time series of the concentration of hs-cTn for participants undergoing secondary coronary angiography 4](#_Toc137822510)

[Figure S2 individual time series of the concentration of hs-cTnT measured by the Elecsys hs-cTnT assay 5](#_Toc137822511)

[Figure S3 individual time series of the concentration of hs-cTnI measured by the Atellica hs-cTnI 6](#_Toc137822512)

[Figure S4 individual time series of the concentration of hs-cTnI measured by the Alinity hs-cTnI assay 7](#_Toc137822513)

[Figure S5 individual time series of the concentration of hs-cTnI measured by the Vitros hs-cTnI assay 8](#_Toc137822514)

[Figure S6 individual time series of the concentration of hs-cTnI measured by the Vista hs-cTnI assay 9](#_Toc137822515)

[Figure S7 individual time series of the concentration of CKMB 10](#_Toc137822516)

[Figure S8 individual time series of the concentration of creatine kinase 11](#_Toc137822517)

[Figure S9 individual time series of the concentration of myoglobin 12](#_Toc137822518)

[Figure S10 individual time series of the concentration of LDH 13](#_Toc137822519)

[Figure S11 individual time series of the concentration of ALT 14](#_Toc137822520)

[Figure S12 individual time series of the concentration of CRP 15](#_Toc137822521)

[Figure S13 trajectories of concentrations as a percentage of the median maximum concentration 16](#_Toc137822522)

[Figure S14 trajectories of concentrations as a percentage of the median maximum concentration 17](#_Toc137822523)

[Figure S15 plots of the median ratio of concentration divided by the upper reference level 18](#_Toc137822524)

[Figure S16 trajectories of hs-cTn concentrations as a percentage of the max concentration 20](#_Toc137822525)

[Figure S17 Bar plot showing when study participants reached their peak concentration 22](#_Toc137822526)

[Figure S18 plots of the decay of cardiac biomarkers reaching a peak within 24 hours 24](#_Toc137822527)

[Figure S19 sensitivity analysis of effect of acute inflammatory response for time to peak from PCI 25](#_Toc137822528)

[Figure S20 26](#_Toc137822529)

[Panel A 26](#_Toc137822530)

[Panel B 26](#_Toc137822531)

[Panel C 27](#_Toc137822532)

[Panel D 28](#_Toc137822533)

##

## Information on upper reference levels and stability of biomarkers

The upper reference level (URL) of the Alinity I STAT high-sensitivity (hs) cardiac troponin (cTn) I assay was 15.6 ng/l for women and 34.2 ng/l for men. The URL of the Dimension Vista hs-cTnI assay was 53.7 ng/l for women and 78.5 ng/l for men. The URL of the Vitros hs-cTnI assay was 9 ng/l for women and 13 ng/l for men. The URL of the Atellica hs-cTnI assay was 34.11 ng/l for women and 53.48 ng/l for men. The URL of the Elecsys hs-cTnT assay was 9 ng/l for women and 16.8 ng/l for men. The URL for LDH was 255 u/l for persons ≤69 years of age and 205 u/l for persons >69 years of age. The URL for ALT was 45 u/l for women and 70 u/l for men. The URL for CRP was 3 mg/l. The URL for myoglobin was 49 µg/l for women and 77 µg/l for men. The URL for CKMB was 4 µg/l for women and 7 µg/l for men. The URL for CK was 210 u/l for women and 270 u/l for men > 49 years of age and 400 u/l for men ≤49 years of age.

The sex-specific URLs for hs-cTn assays were defined by the manufacturers and is specified in the insert for each assay.

The inserts for the Alinity hs-cTnI assay, the Atellica hs-cTnI assay and the Vista hs-cTnI assay state that plasma can be stored at room temperature for 8 hours and at 2-8 degrees Celsius for 24 hours prior to centrifugation. The insert for the Vitros hs-cTnI assay state that the plasma can be stored at room temperature for 8 hours and at 2-8 degrees Celsius for 2 days. The insert for the Elecsys hs-cTnT state that plasma can be stored at 2-8 degrees Celsius for 24 hours prior to centrifugation.

We have previously tested cTn stability for the Vista hs-cTnI assay which yielded the following results.

| Sample number | Centrifugation and analysis of sample at time 0 | Centrifugation and analysis of sample 8 hours later at room temperature |
| --- | --- | --- |
| 1 | 4,9 | 4,9 |
| 2 | 154 | 156,6 |
| 3 | 6,1 | 4,8 |
| 4 | 5,4 | 6,8 |
| 5 | 4,9 | 4,3 |
| 6 | 6 | 6 |
| 7 | 153 | 146,6 |
| 8 | 3,7 | 3,1 |
| 9 | 7,7 | 8 |
| 10 | 620 | 606,8 |
| 11 | 4,5 | 4,1 |
| 12 | 1140 | 1105,7 |
| 13 | 7,6 | 7,8 |
| 14 | 8,1 | 8,6 |
| 15 | 129 | 139,8 |
| 16 | 24 | 23,8 |
| 17 | 25 | 24,5 |
| 18 | 5,3 | 5,5 |
| 19 | 5,8 | 6 |
| 20 | 29 | 27,2 |
| 21 | 88 | 88,6 |
| 22 | 14 | 13,6 |
| 23 | 44 | 43,2 |
| 24 | 37 | 34,4 |
| 25 | 36 | 30,6 |
| 26 | 4 | 3,4 |

## individual time series of the concentration of hs-cTn for participants undergoing secondary coronary angiography


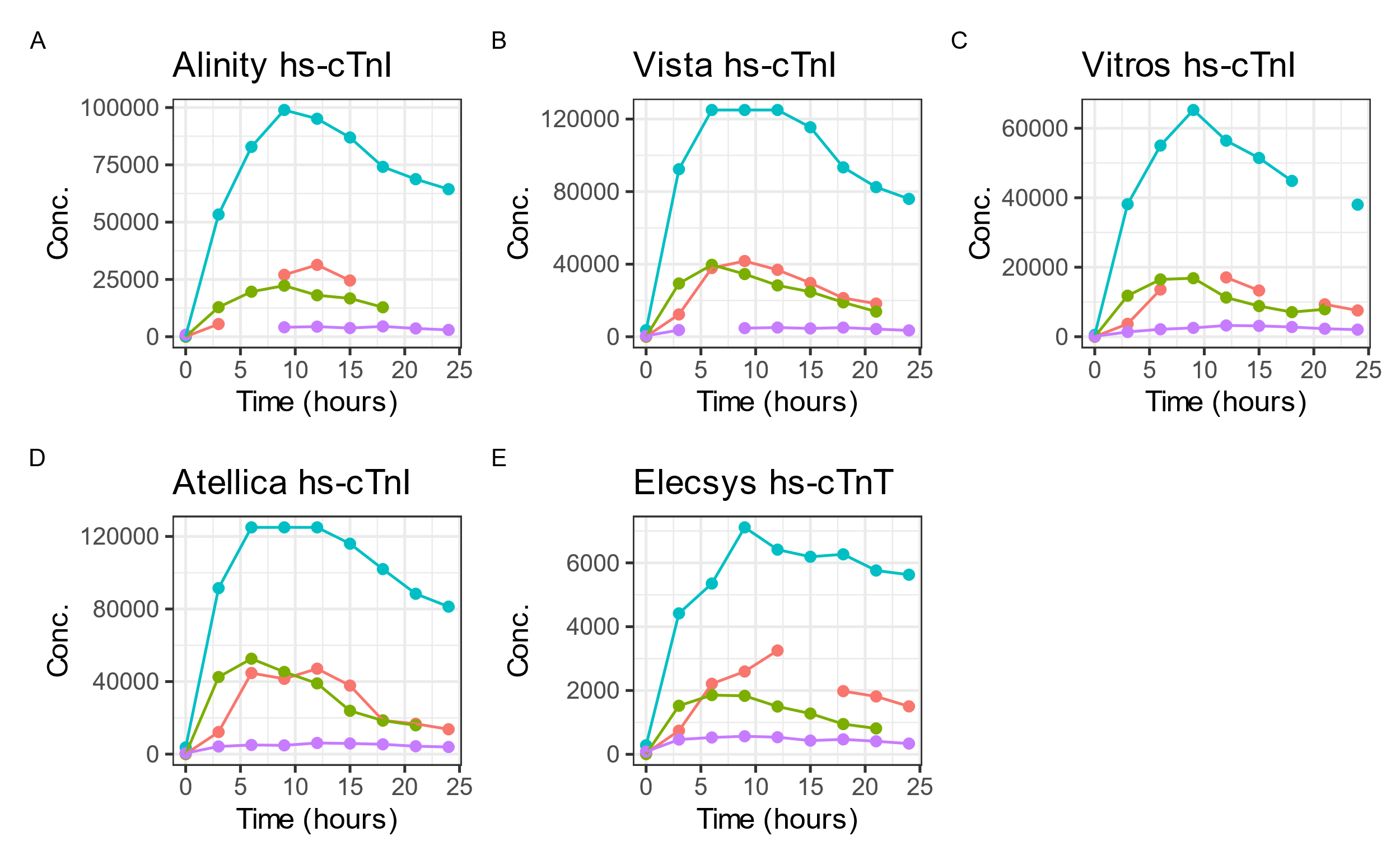


Individual time series of high-sensitivity (hs) cardiac troponin (cTn) concentrations. Time is presented in hours from acute revascularization to 24 hours after. Each line corresponds to one study participant. Panel A: Alinity hs-cTnI assay. Panel B: Vista hs-cTnI assay. Panel C: Vitros hs-cTnI assay. Panel D: Atellica hs-cTnI assay. Panel E: Elecsys hs-cTnT assay. Conc.: Concentration.

## individual time series of the concentration of hs-cTnT measured by the Elecsys hs-cTnT assay


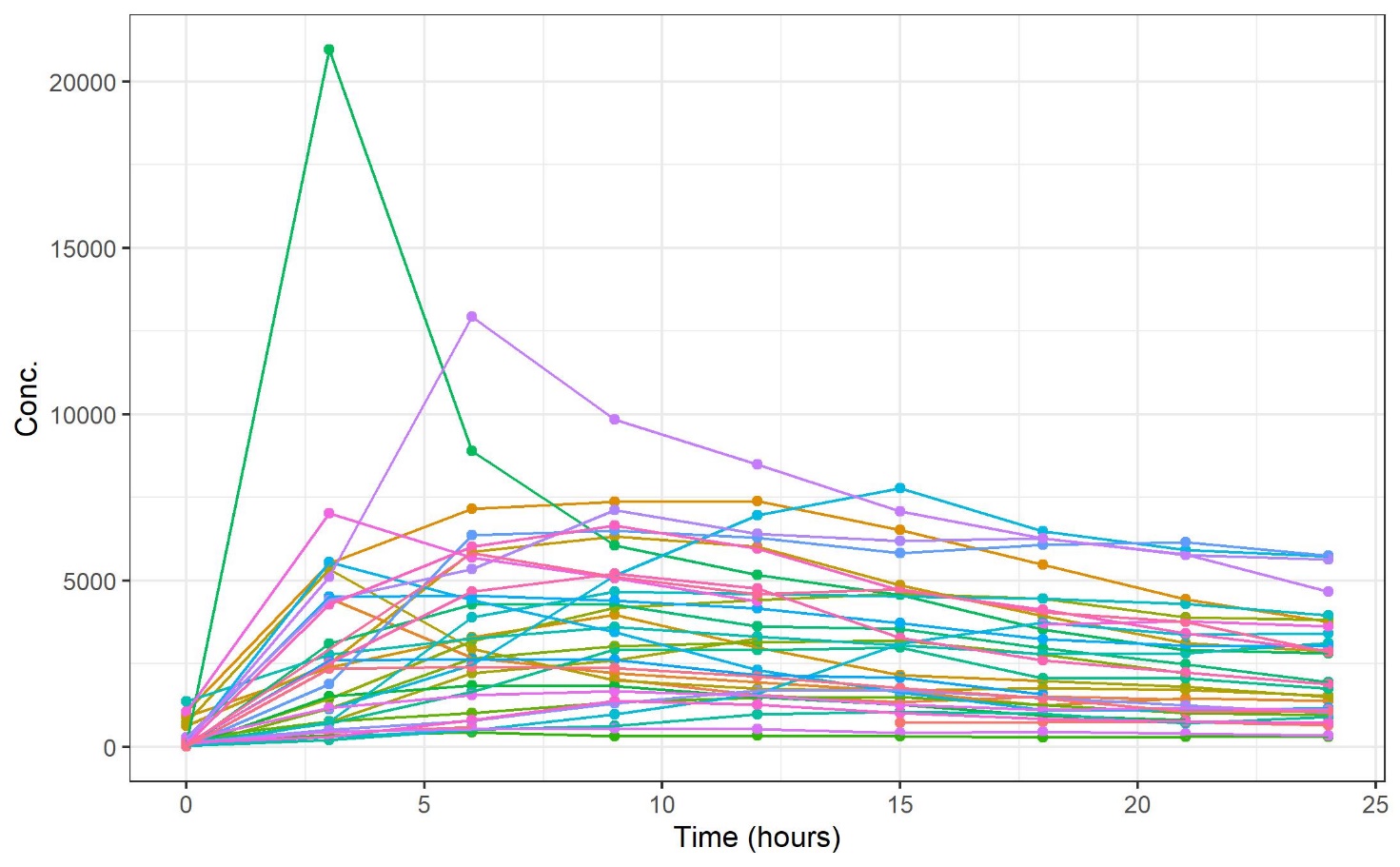


Individual time series of the concentration of high-sensitivity (hs) cardiac troponin (cTn) T measured by the Elecsys hs-cTnT assay in ng/l. Time is presented in hours from acute revascularization to 24 hours after. Each line corresponds to one study participant. Conc.: Concentration.

## individual time series of the concentration of hs-cTnI measured by the Atellica hs-cTnI


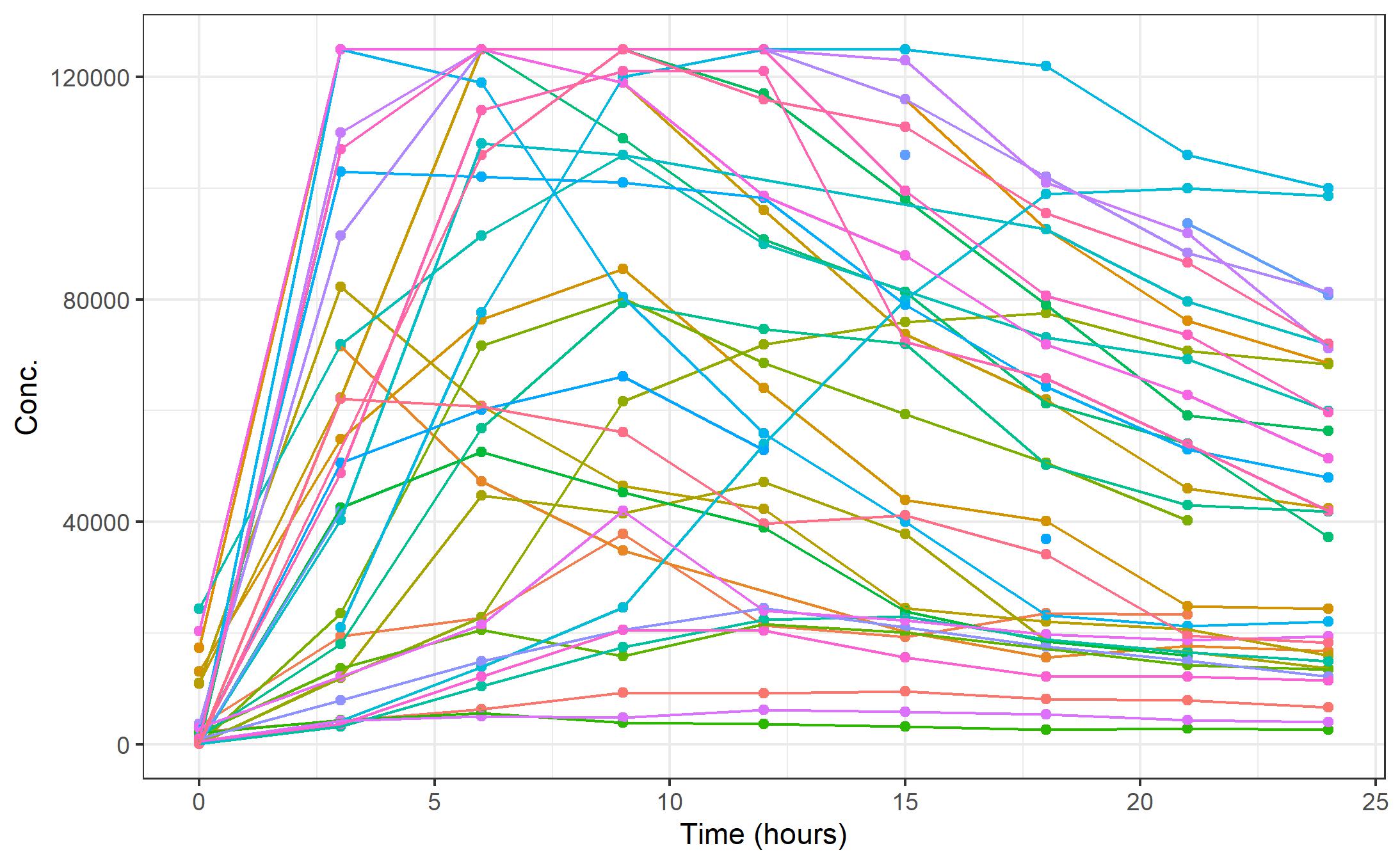


Individual time series of the concentration of high-sensitivity (hs) cardiac troponin (cTn) I measured by the Atellica hs-cTnI assay in ng/l. Time is presented in hours from acute revascularization to 24 hours after. Each line corresponds to one study participant. Conc.: Concentration.

## individual time series of the concentration of hs-cTnI measured by the Alinity hs-cTnI assay


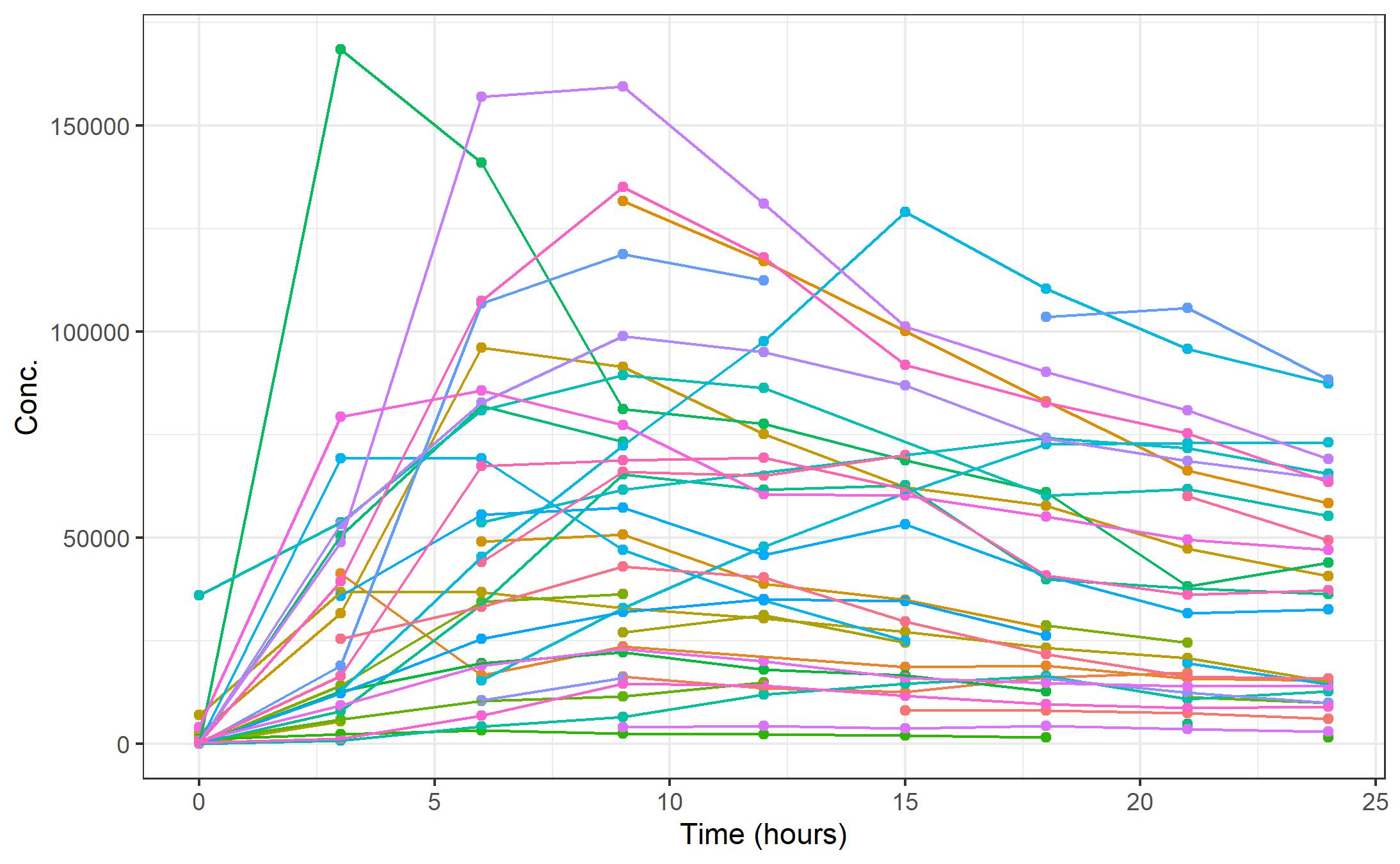


Individual time series of the concentration of high-sensitivity (hs) cardiac troponin (cTn) I measured by the Alinity hs-cTnI assay in ng/l. Time is presented in hours from acute revascularization to 24 hours after. Each line corresponds to one study participant. Conc.: Concentration.

## individual time series of the concentration of hs-cTnI measured by the Vitros hs-cTnI assay


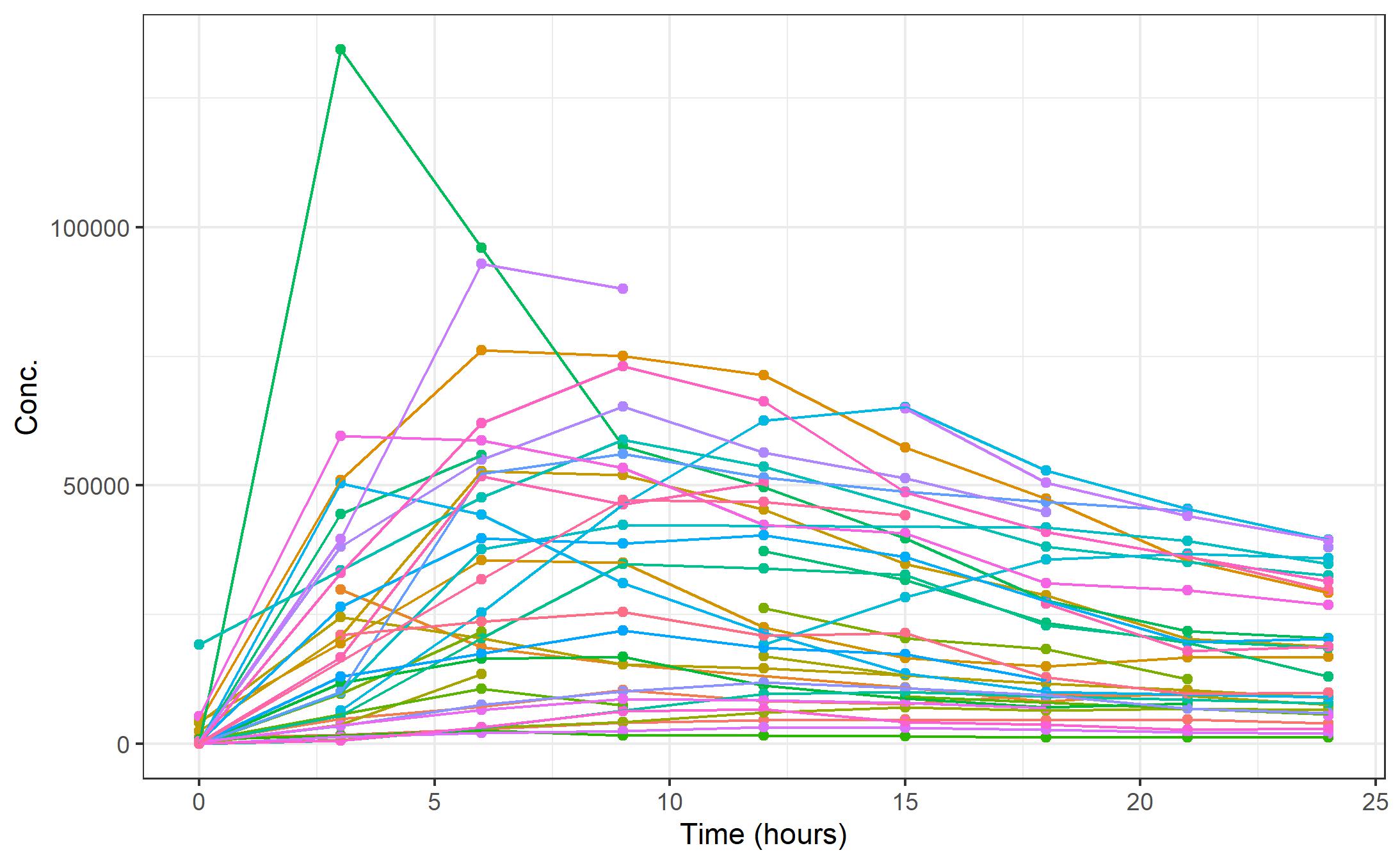


Individual time series of the concentration of high-sensitivity (hs) cardiac troponin (cTn) I measured by the Vitros hs-cTnI assay in ng/l. Time is presented in hours from acute revascularization to 24 hours after. Each line corresponds to one study participant. Conc.: Concentration.

## individual time series of the concentration of hs-cTnI measured by the Vista hs-cTnI assay


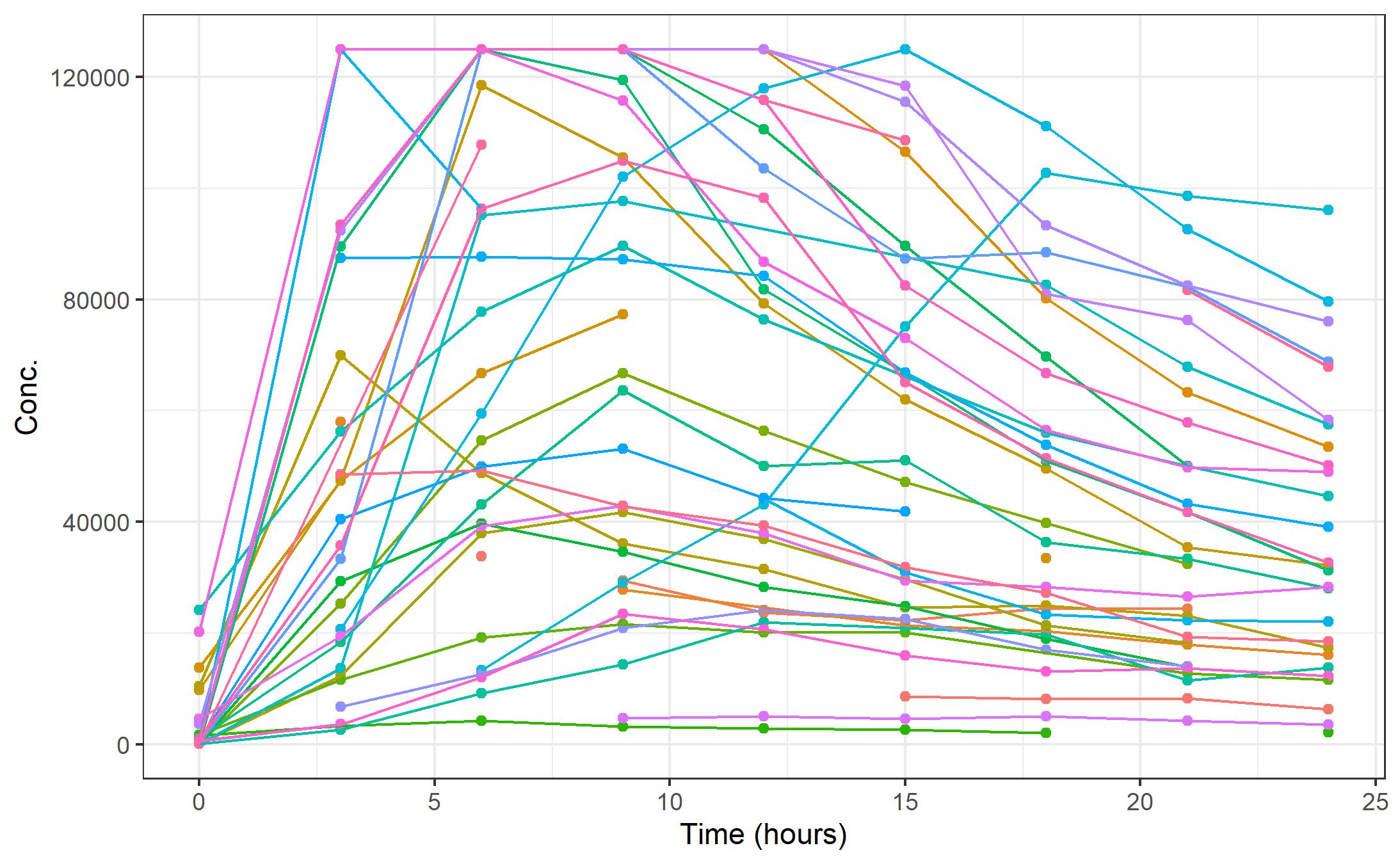


Individual time series of the concentration of high-sensitivity (hs) cardiac troponin (cTn) I measured by the Vista hs-cTnI assay in ng/l. Time is presented in hours from acute revascularization to 24 hours after. Each line corresponds to one study participant. Conc.: Concentration.

## individual time series of the concentration of CKMB


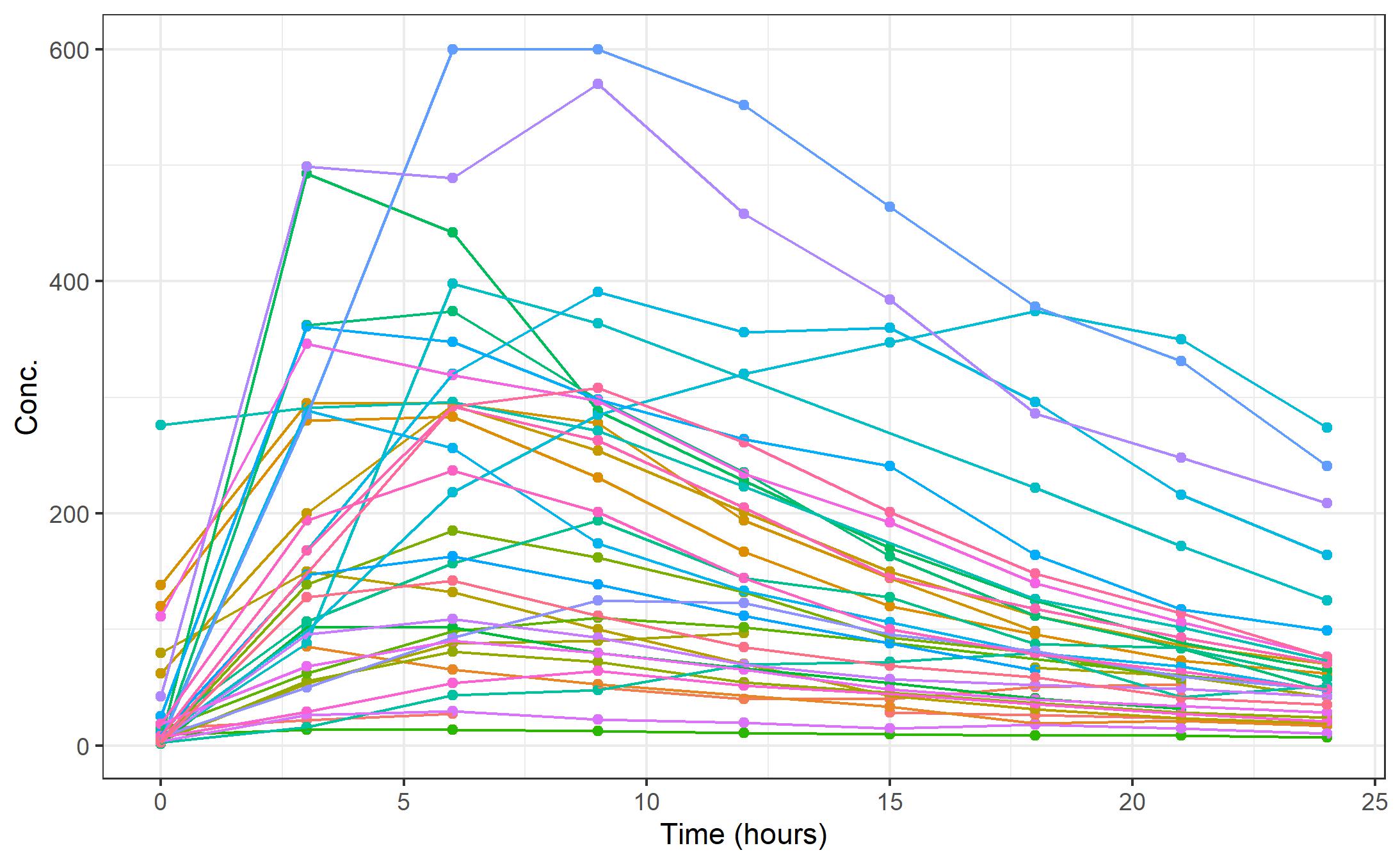


Individual time series of the concentration of creatine kinase MB (CKMB) in µg/l. Time is presented in hours from acute revascularization to 24 hours after. Each line corresponds to one study participant. Conc.: Concentration.

## individual time series of the concentration of creatine kinase


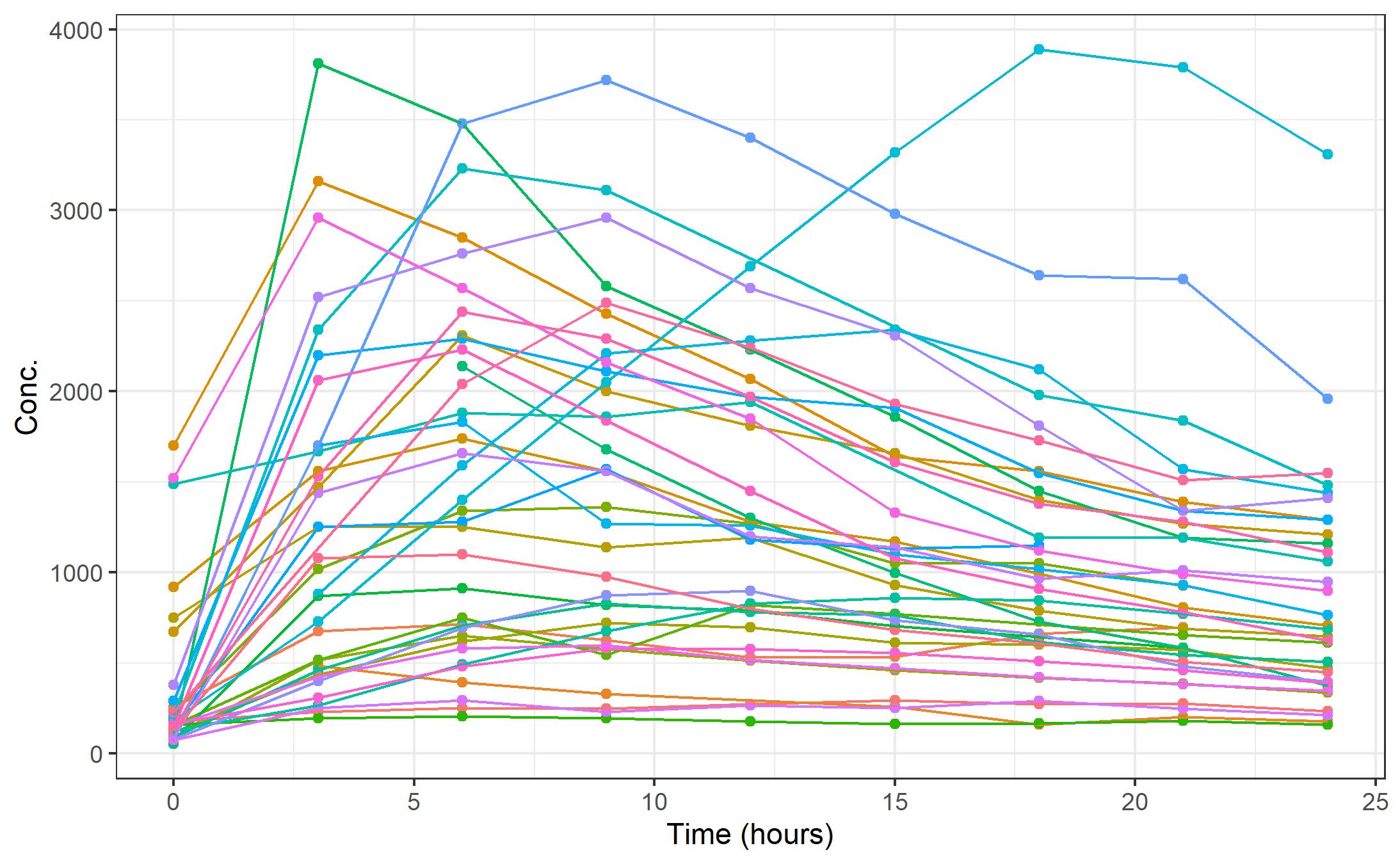


Individual time series of the concentration of creatine kinase in u/l. Time is presented in hours from acute revascularization to 24 hours after. Each line corresponds to one study participant. Conc.: Concentration.

## individual time series of the concentration of myoglobin


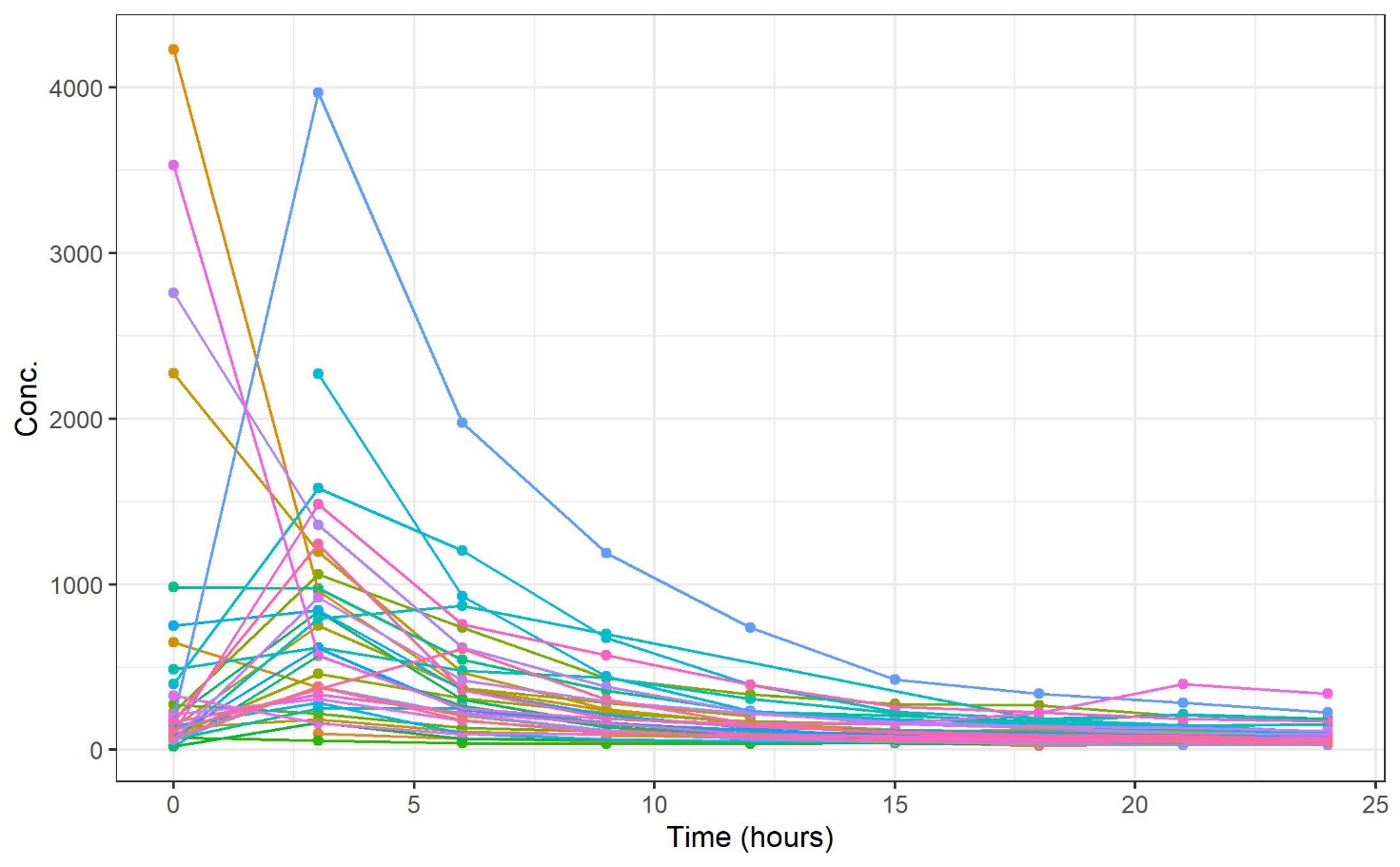


Individual time series of the concentration of myoglobin in µg/l. Time is presented in hours from acute revascularization to 24 hours after. Each line corresponds to one study participant.

## individual time series of the concentration of LDH


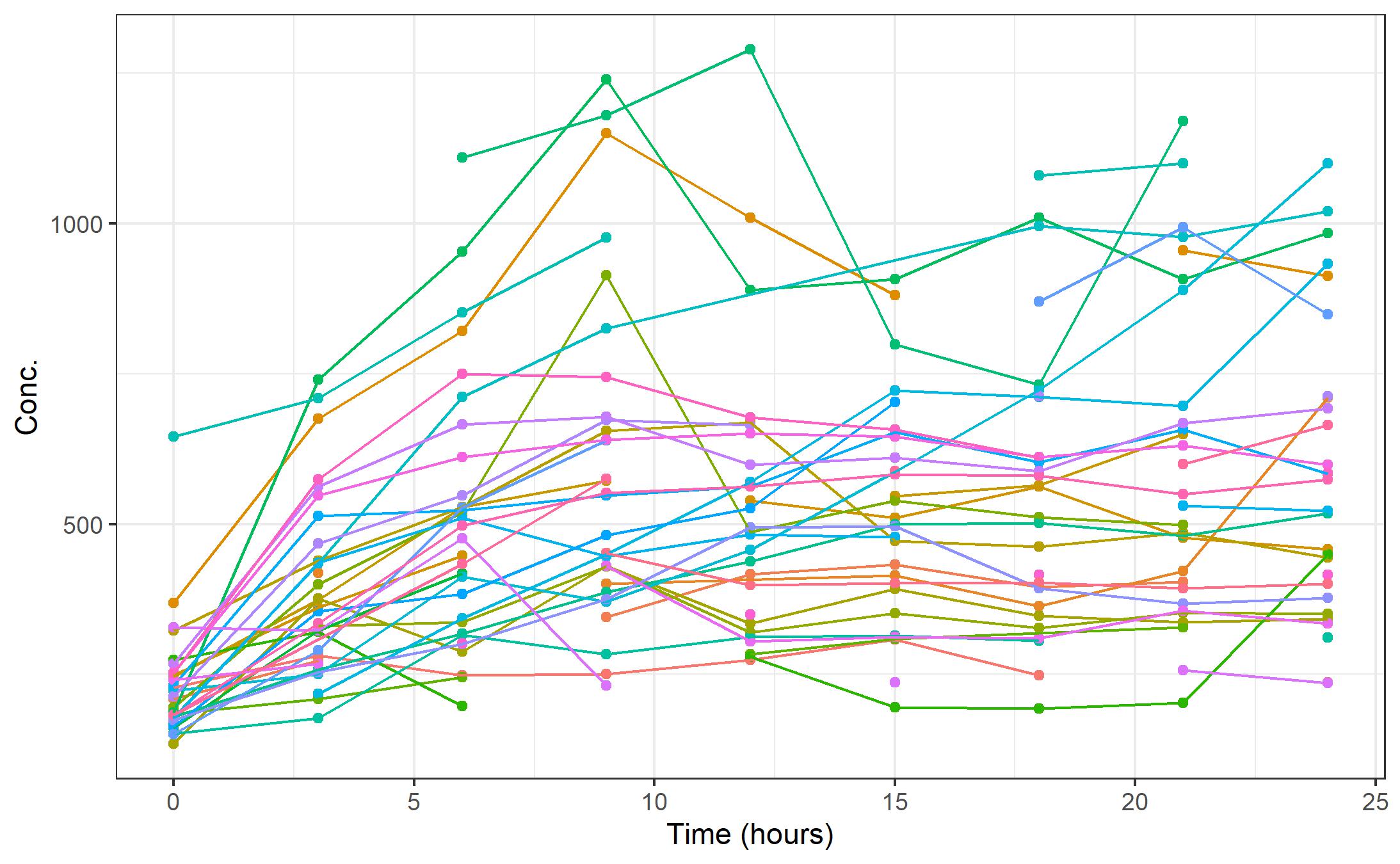


Individual time series of the concentration of lactate dehydrogenase (LDH) in u/l. Time is presented in hours from acute revascularization to 24 hours after. Each line corresponds to one study participant. Conc.: Concentration.

## individual time series of the concentration of ALT


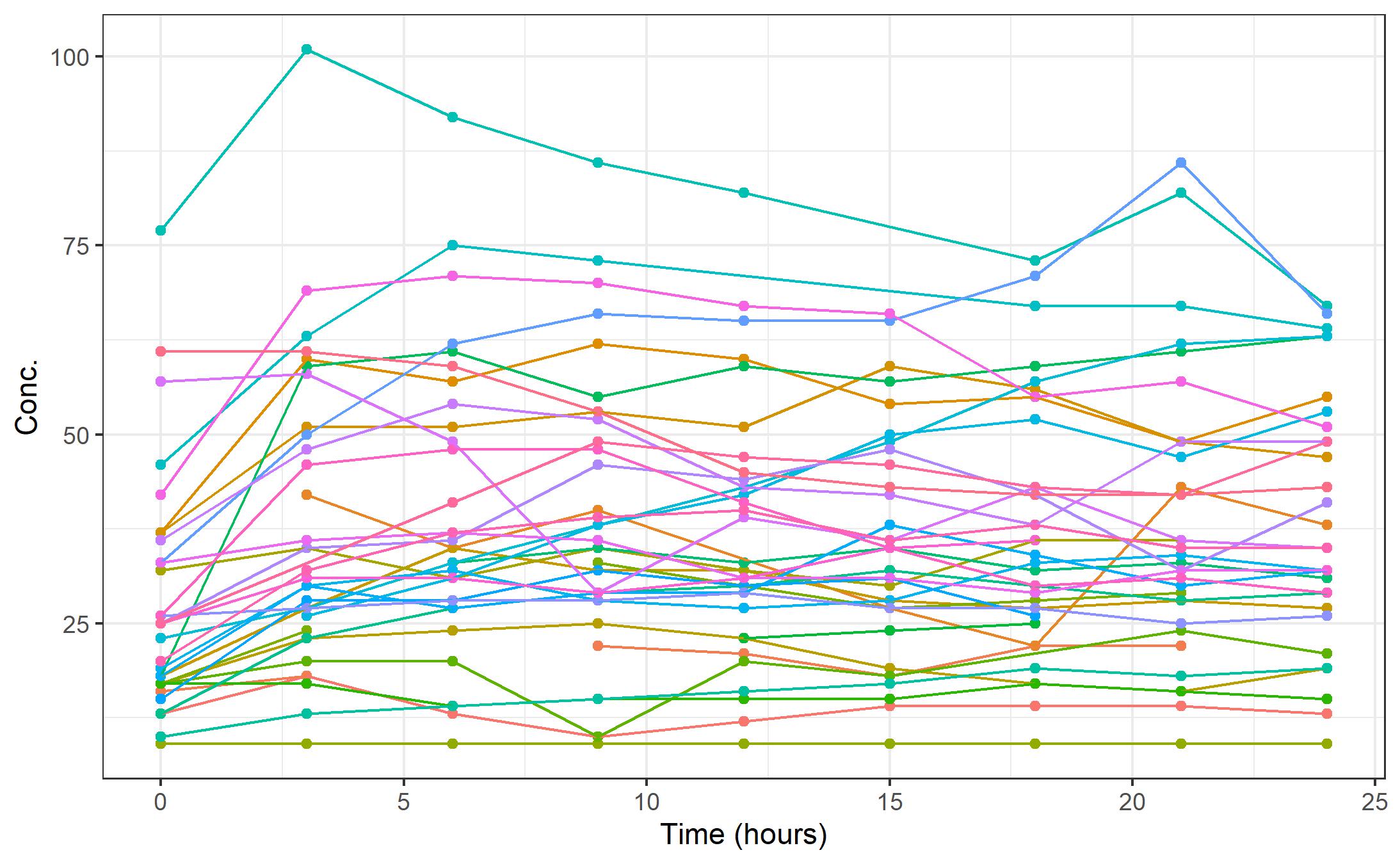


Individual time series of the concentration of alanine transferase (ALT) in u/l. Time is presented in hours from acute revascularization to 24 hours after. Each line corresponds to one study participant. Conc.: Concentration.

## individual time series of the concentration of CRP


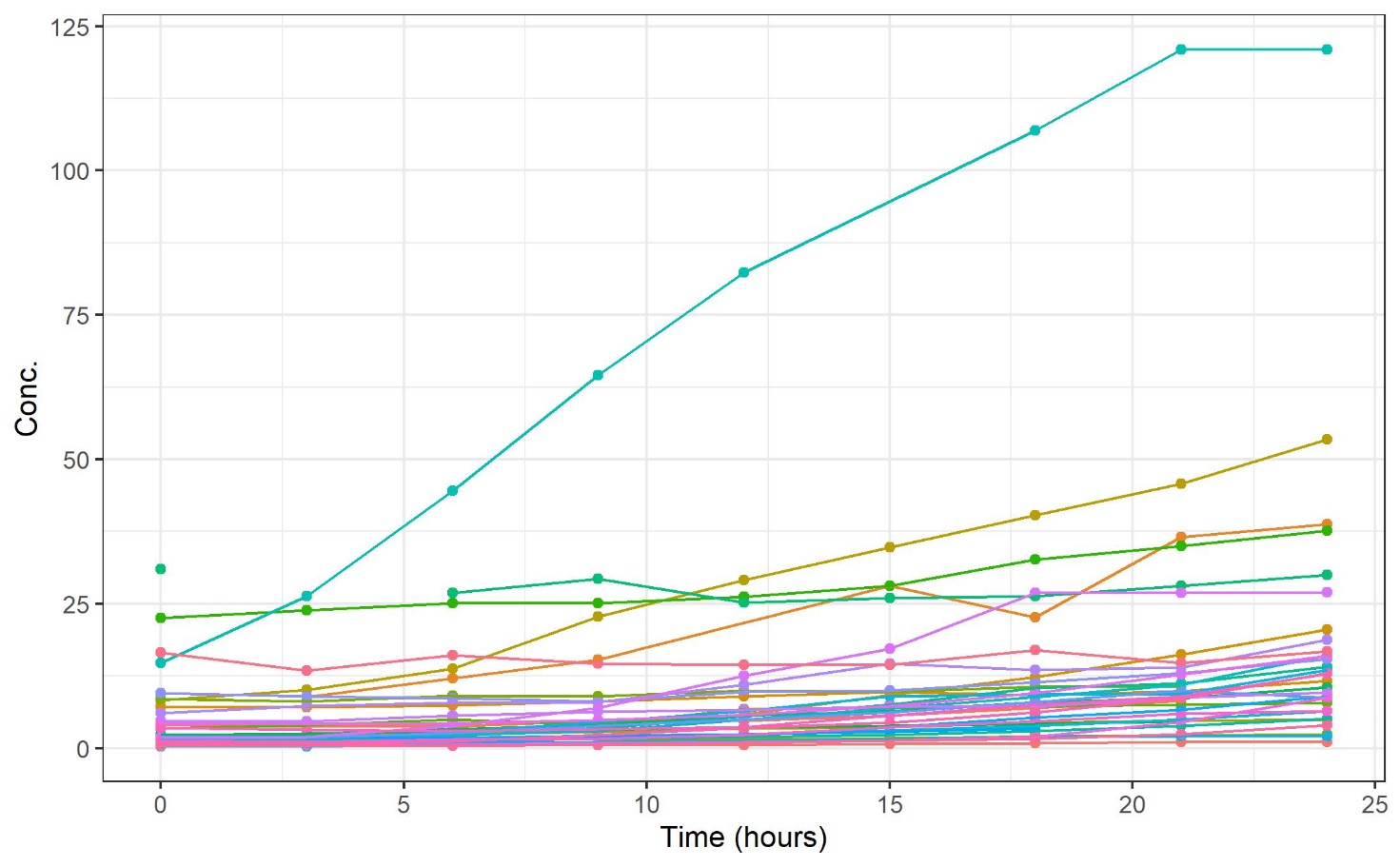


Individual time series of the concentration of C-reactive protein (CRP) in mg/l. Time is presented in hours from acute revascularization to 24 hours after. Each line corresponds to one study participant. Conc.: Concentration.

## trajectories of concentrations as a percentage of the median maximum concentration


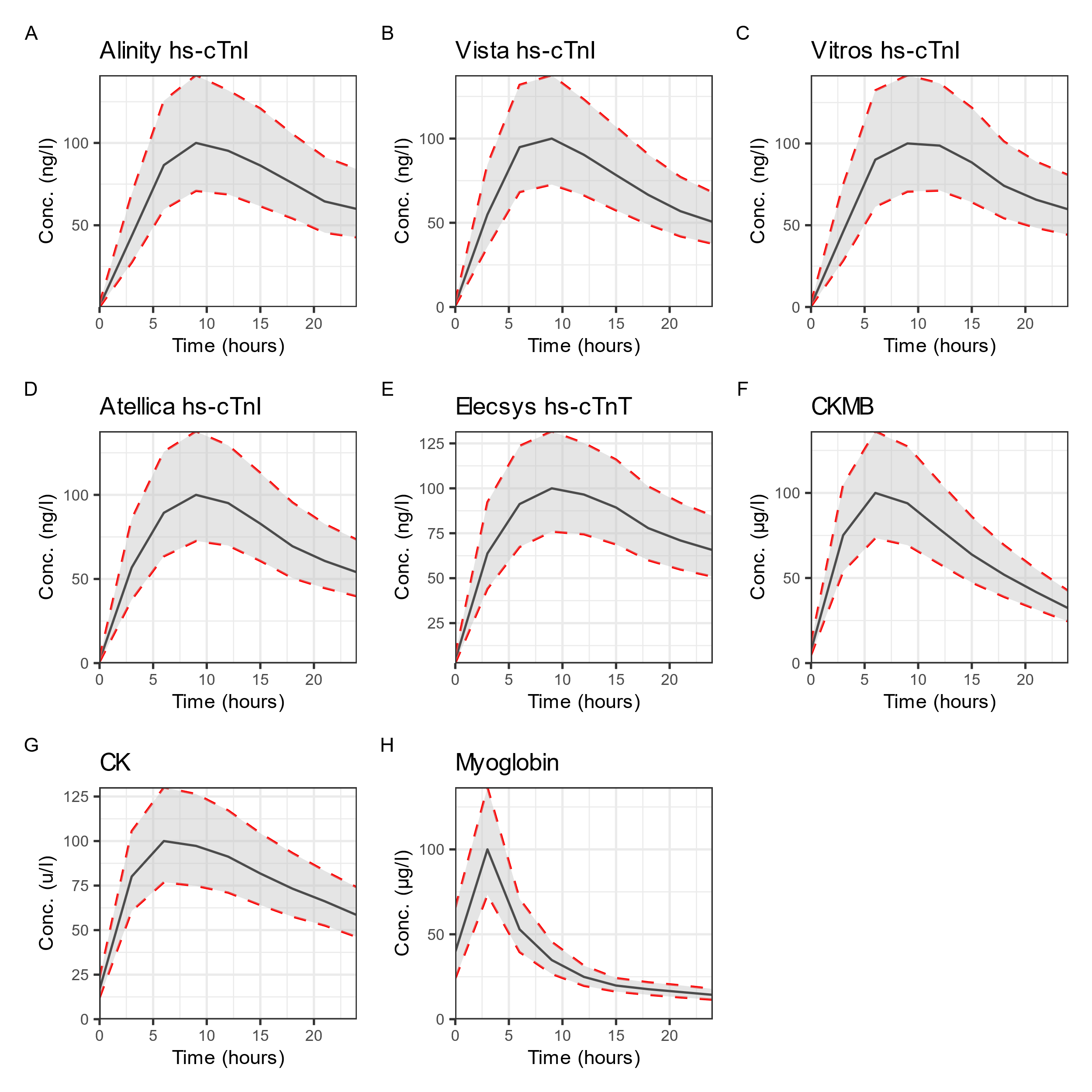


Trajectories of concentrations as a percentage of the median maximum concentration with 95% CI. Time is presented in hours from acute revascularization to 24 hours after. Panel A: Alinity high-sensitivity (hs) cardiac troponin (cTn) I assay. Panel B: Vista hs-cTnI assay. Panel C: Vitros hs-cTnI assay. Panel D: Atellica hs-cTnI assay. Panel E: Elecsys hs-cTnT assay. Panel F: creatine kinase MB. Panel G: creatine kinase. Panel H: myoglobin.

## trajectories of concentrations as a percentage of the median maximum concentration


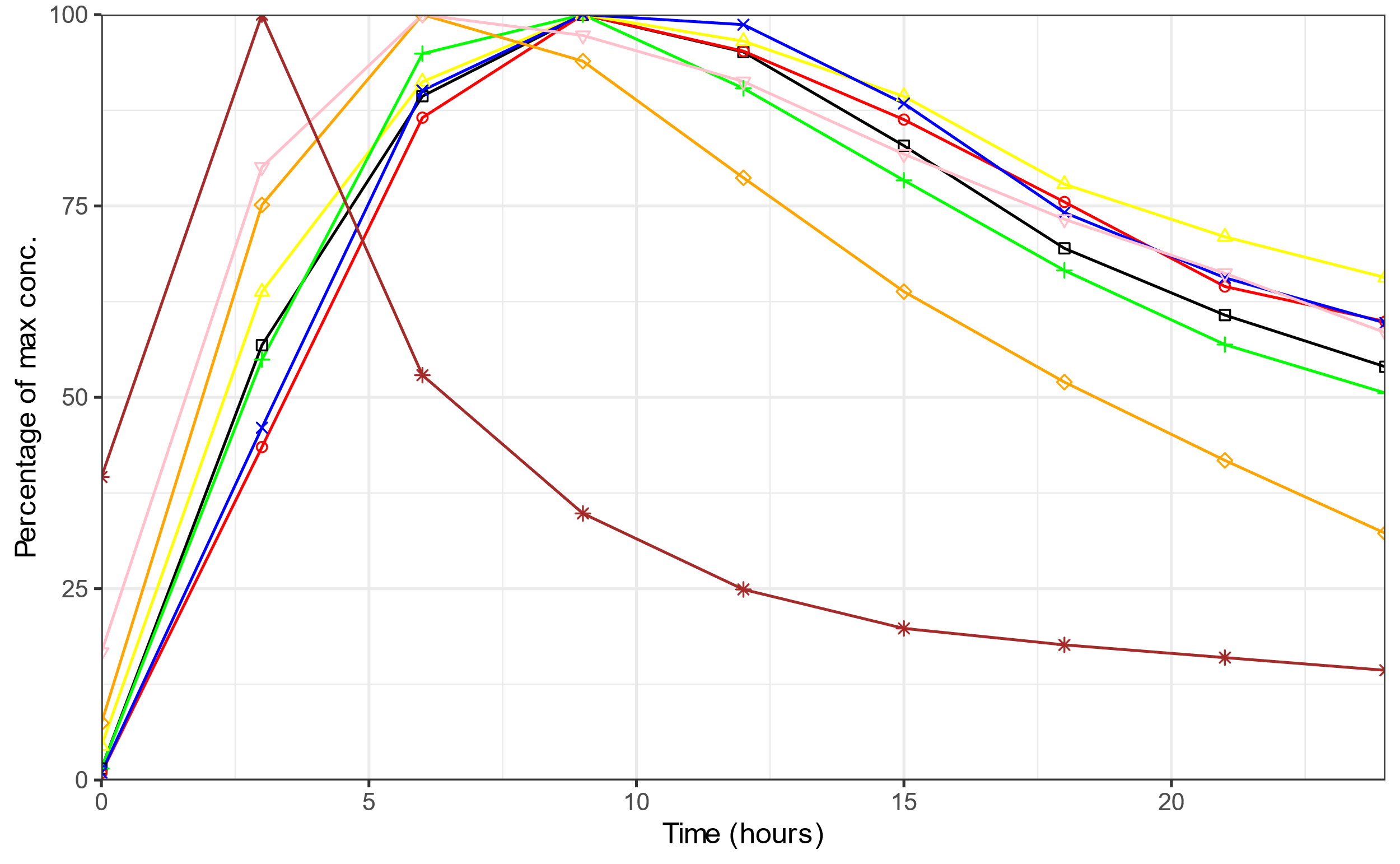


Trajectories of concentrations as a percentage of the median maximum concentration. Time is presented in hours from acute revascularization to 24 hours after. Black (square): Atellica high-sensitivity (hs) cardiac troponin (cTn) I assay. Yellow (triangle point up): Elecsys hs-cTnT assay. Green (plus): Vista hs-cTnI assay. Blue (cross): Vitros hs-cTnI assay. Red (circle): Alinity hs-cTnI assay. Orange (diamond): creatine kinase MB (CKMB). Brown (star): Myoglobin. Pink (triangle point down): creatine kinase. Conc.: Concentration.

## plots of the median ratio of concentration divided by the upper reference level


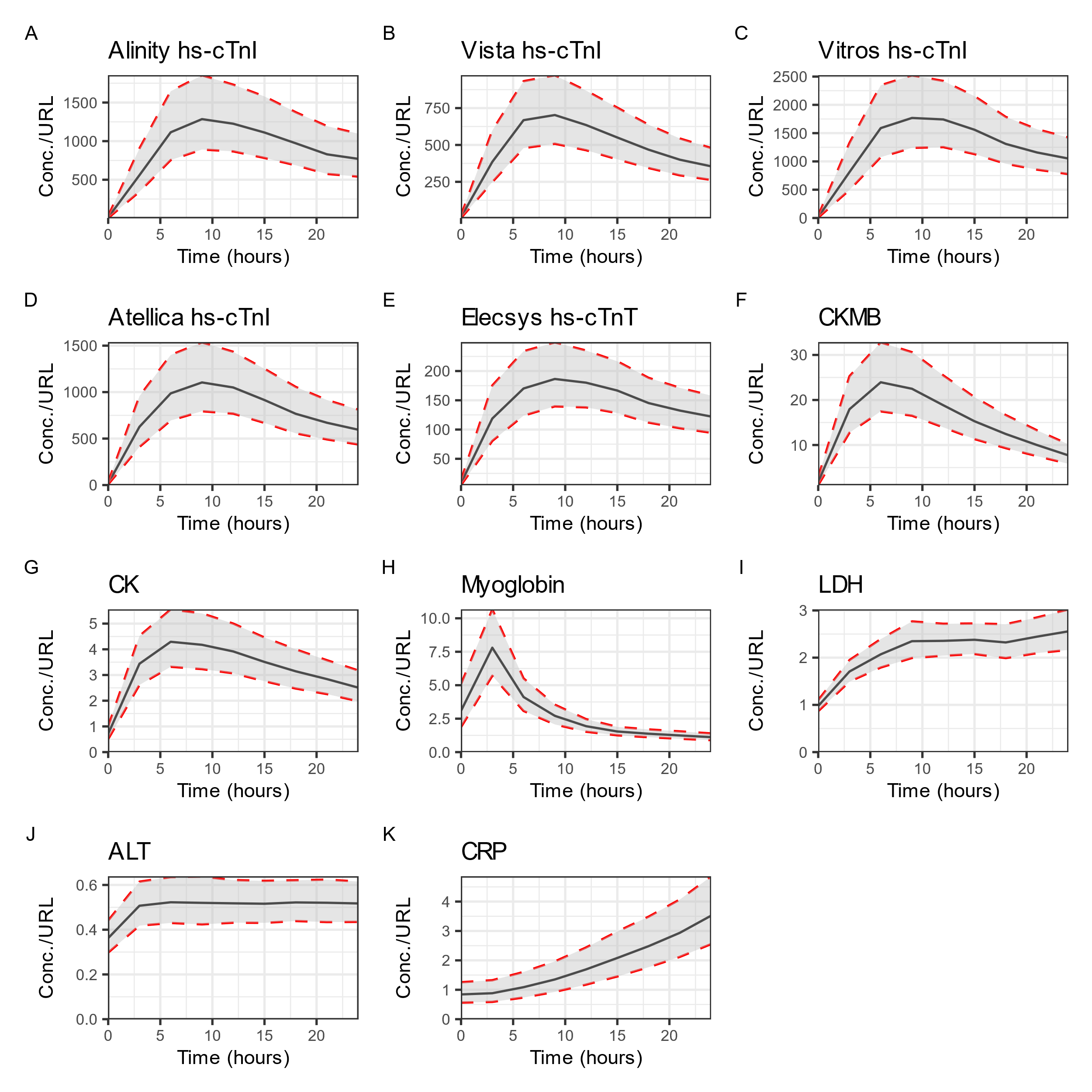


Plots of the median ratio of concentration divided by the upper reference level (URL) with 95% CI. Time is presented in hours from acute revascularization to 24 hours after. The URLs are stated in the supplementary appendix. Panel A: Alinity high-sensitivity (hs) cardiac troponin (cTn) I assay in ng/l. Panel B: Vista hs-cTnI assay in ng/l. Panel C: Vitros hs-cTnI assay in ng/l. Panel D: Atellica hs-cTnI assay in ng/l. Panel E: Elecsys hs-cTnT assay in ng/l. Panel F: creatine kinase (CK) MB in µg/l. Panel G: CK in U/l. Panel H: myoglobin in µg/l. Panel I: lactate dehydrogenase (LDH) in u/l. Panel J: alanine transaminase (ALT) in u/l. Panel K: C-reactive protein (CRP) in mg/l. Conc./URL: Concentration/upper reference level.

## trajectories of hs-cTn concentrations as a percentage of the max concentration


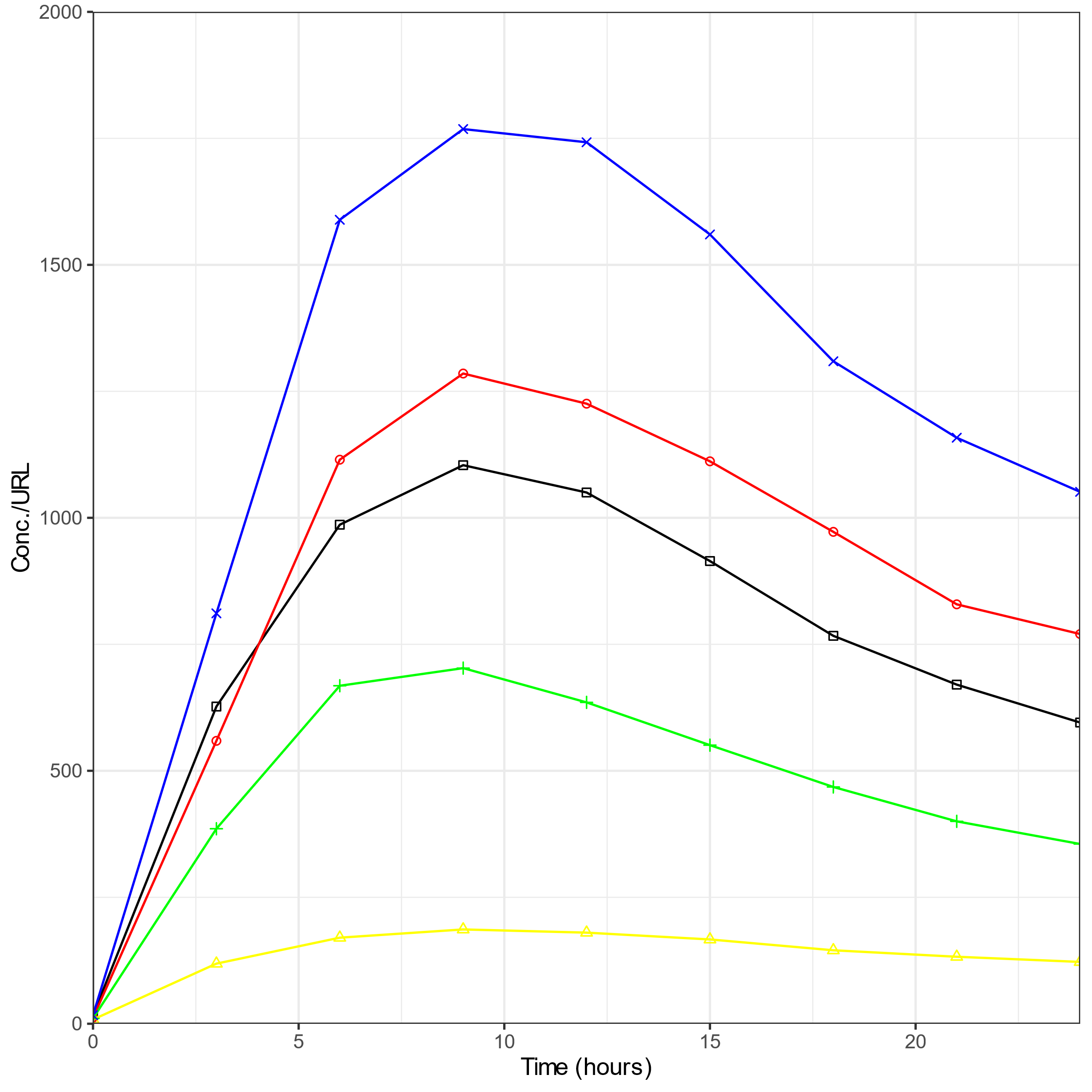


The median ratio of concentration divided by the upper reference level of high-sensitivity (hs) cardiac troponin (cTn). Time is presented in hours from acute revascularization to 24 hours after. Black (square): Alinity hs-cTnI assay. Yellow (triangle point up): Elecsys hs-cTnT assay. Green (plus): Vista hs-cTnI assay. Blue (cross): Vitros hs-cTnI assay. Red (circle): Alinity hs-cTnI assay. Conc./URL: Concentration/upper reference level.

## Bar plot showing when study participants reached their peak concentration


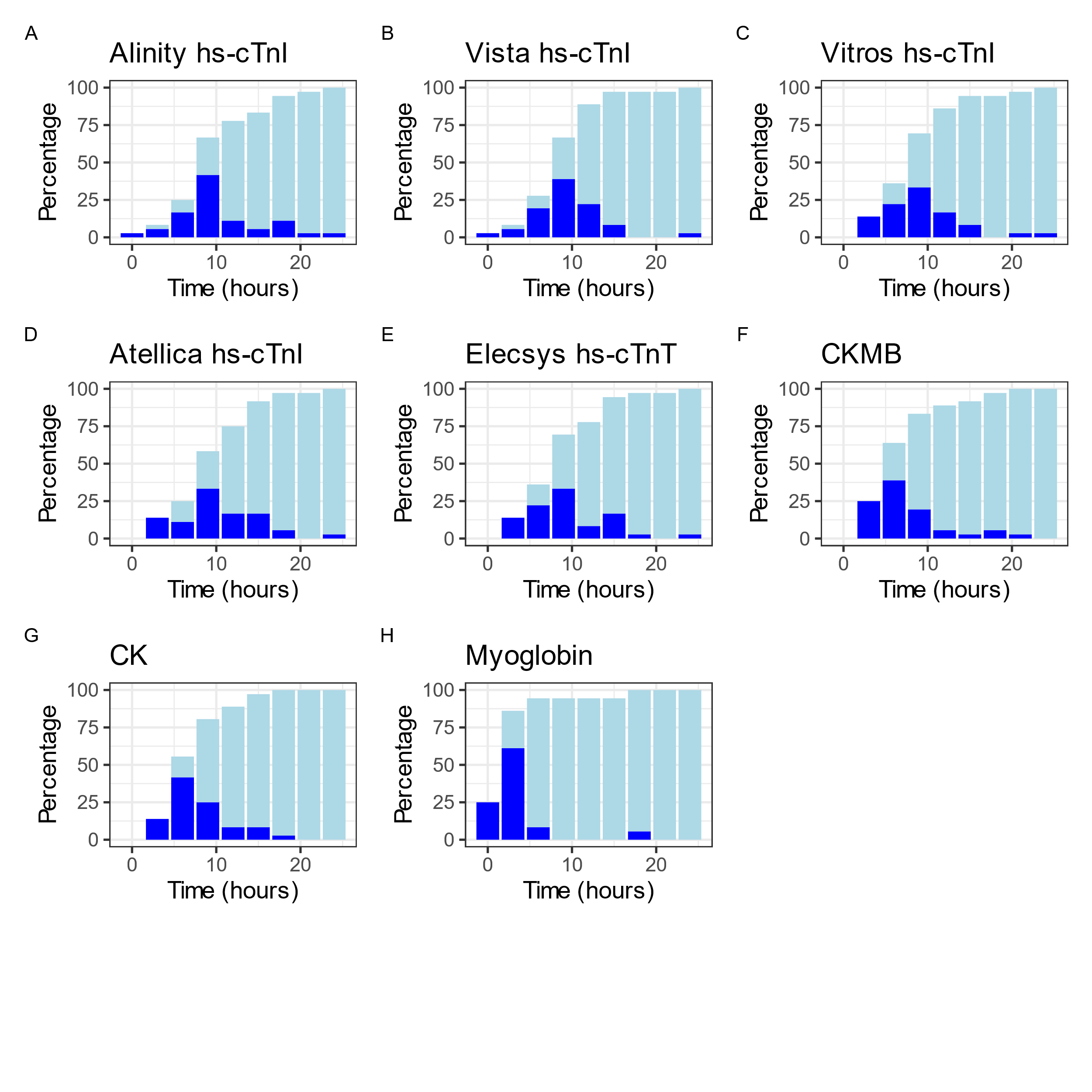


Bar plot showing when study participants reached their peak concentration. Biomarkers reaching a peak concentration denoted by a rise and fall are represented. Time is presented in hours from acute revascularization to 24 hours after. Dark blue: participants’ peak concentration. Both light and dark blue: cumulative percentage. Panel A: Alinity high-sensitivity (hs) cardiac troponin (cTn) I assay in ng/l. Panel B: Vista hs-cTnI assay in ng/l. Panel C: Vitros hs-cTnI assay in ng/l. Panel D: Atellica hs-cTnI assay in ng/l. Panel E: Elecsys hs-cTnT assay in ng/l. Panel F: creatine kinase (CK) MB in µg/l. Panel G: CK in U/l. Panel H: myoglobin in µg/l.

## plots of the decay of cardiac biomarkers reaching a peak within 24 hours


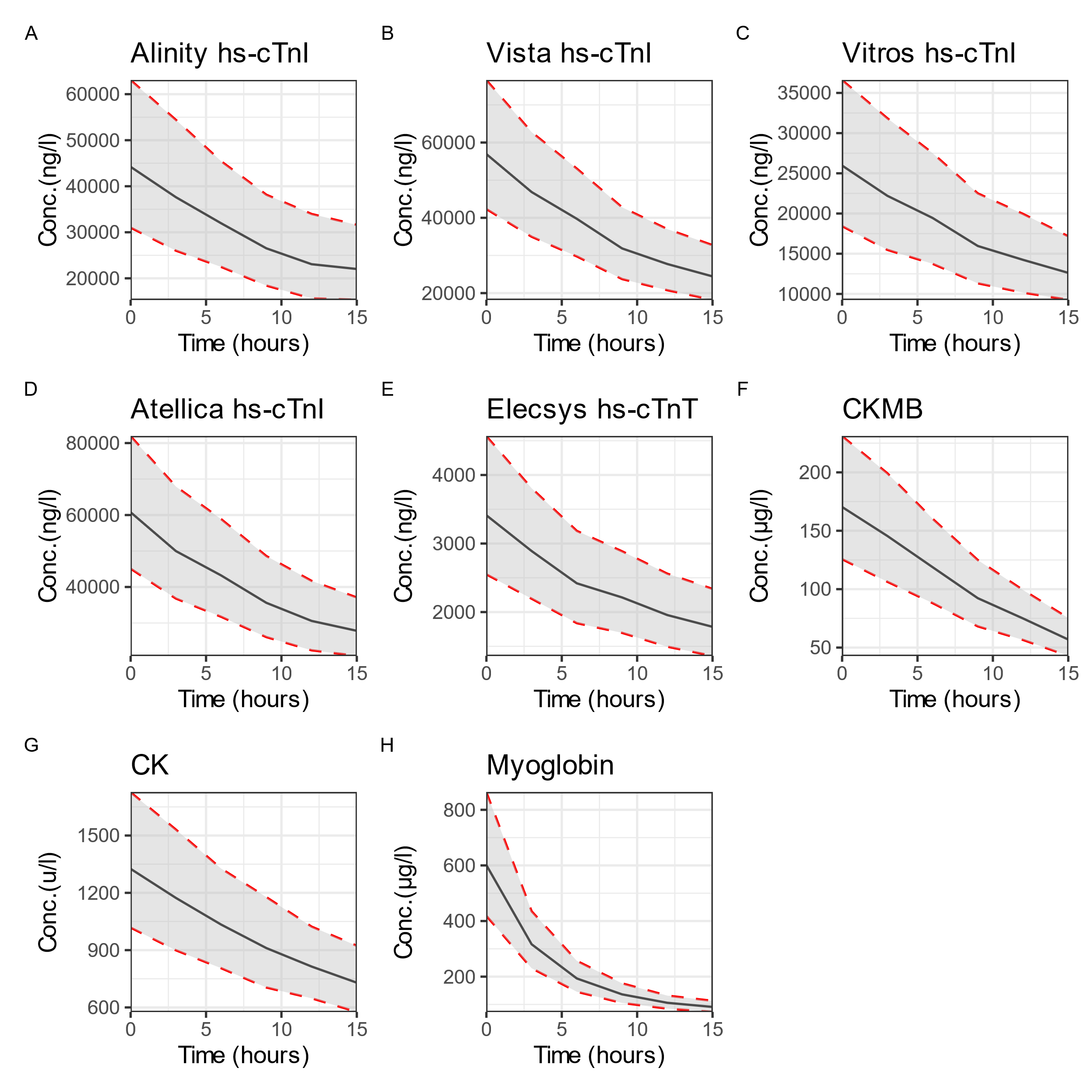


Plots of the decay of cardiac biomarkers reaching a peak within 24 hours with 95% CI. Time is presented in hours from peak concentration to 15 hours after. Panel A: Alinity high-sensitivity (hs) cardiac troponin (cTn) I assay in ng/l. Panel B: Vista hs-cTnI assay in ng/l. Panel C: Vitros hs-cTnI assay in ng/l. Panel D: Atellica hs-cTnI assay in ng/l. Panel E: Elecsys hs-cTnT assay in ng/l. Panel F: creatine kinase (CK) MB in µg/l. Panel G: CK in U/l. Panel H: myoglobin in µg/l. Conc.: Concentration.

## Figure S19 sensitivity analysis of effect of acute inflammatory response for time to peak from PCI

| **Biomarker** | **Median time from PCI to peak concentration in hours (95%-CI)** |
| --- | --- |

*
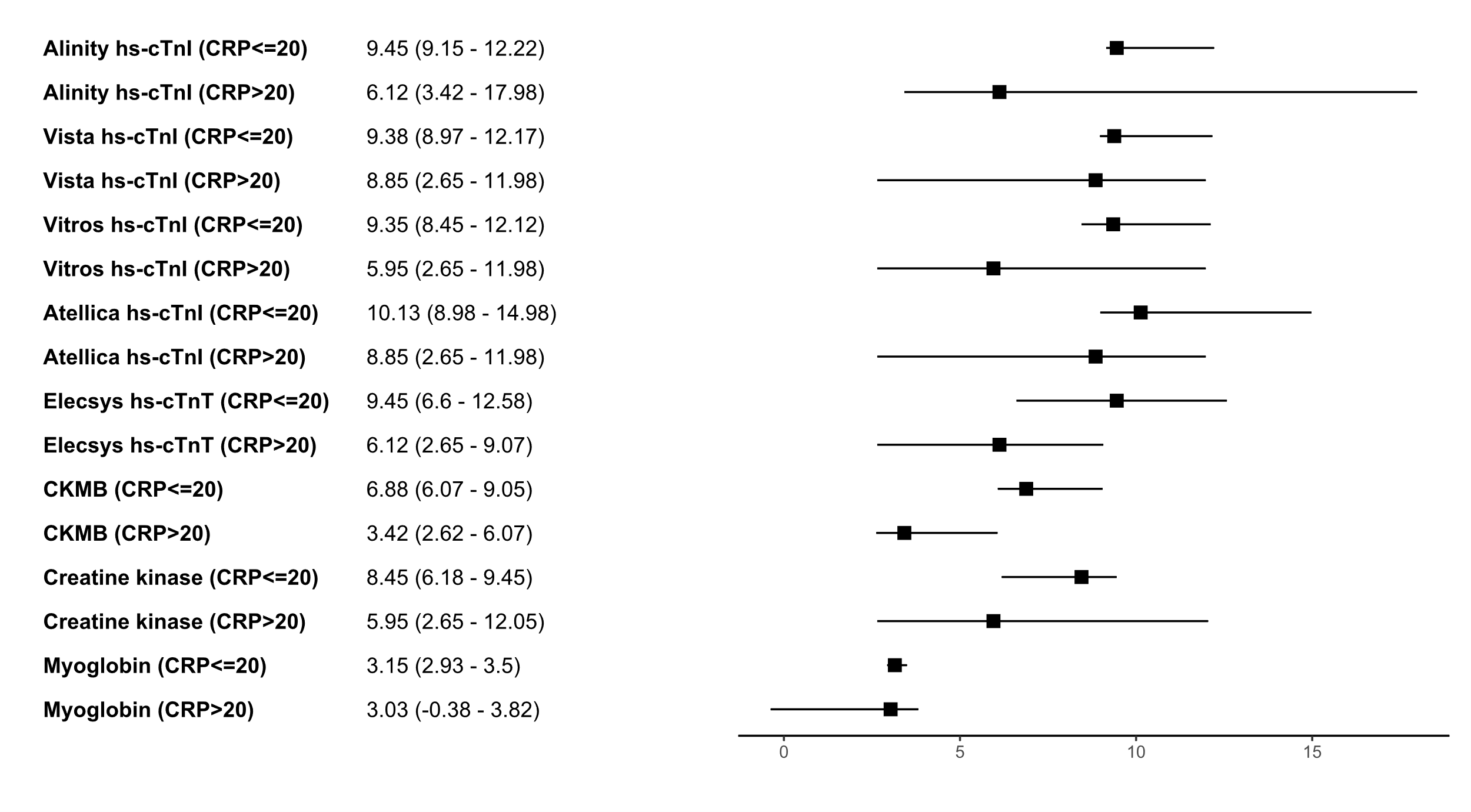
*

Forrest plots of time to peak for high sensitivity (hs) cardiac troponins (cTn), creatine kinase (CK), CKMB, and myoglobin stratified according to CRP<=20 (n=29) and CRP>20 (n=7). Time is in hours.

## Figure S20

###
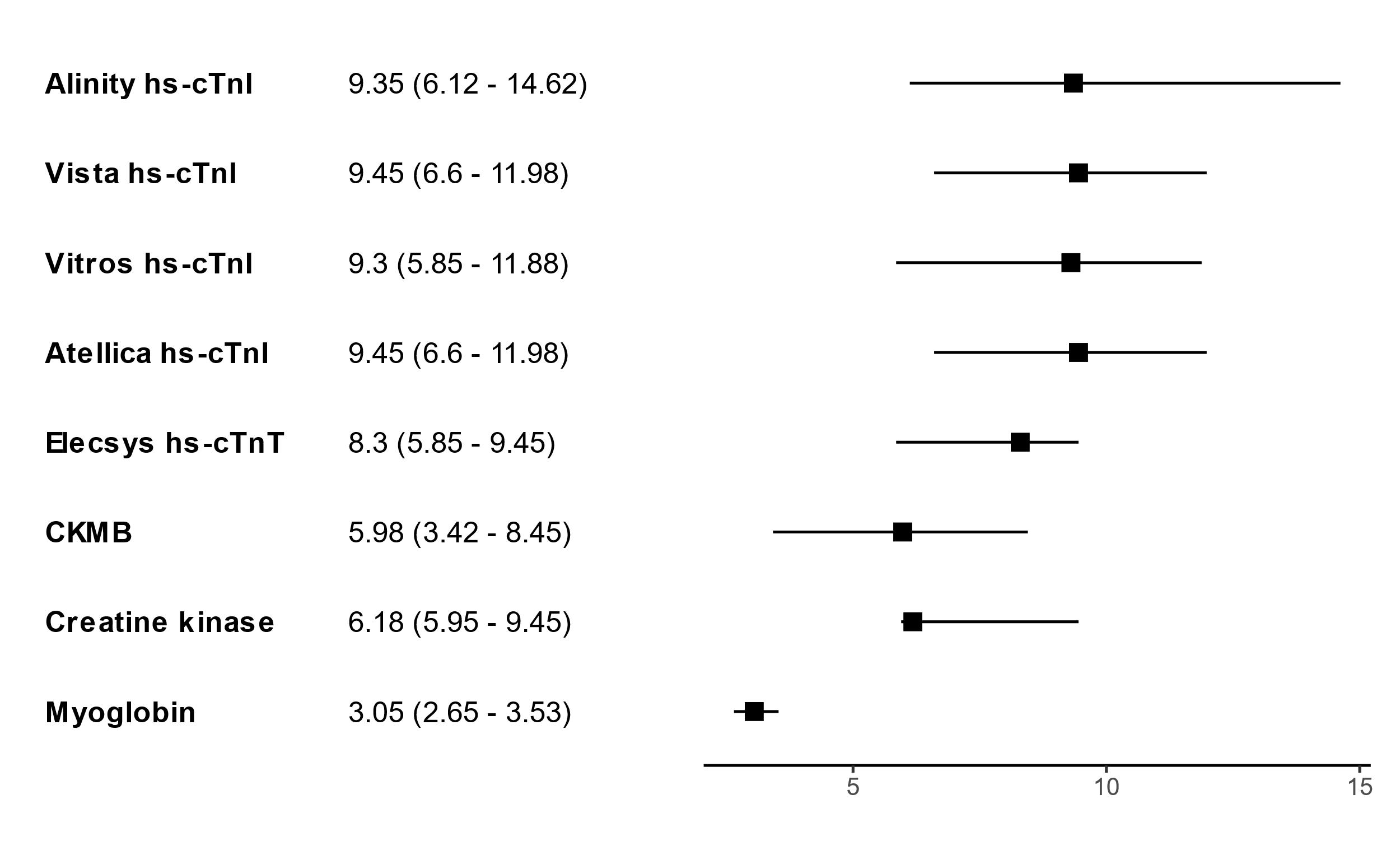
Panel A

| **Biomarker** | **Median time from PCI to peak concentration in hours (95%-CI)** |
| --- | --- |

### Panel B

| **Biomarker** | **Median time from symptoms onset to peak concentration in hours (95%-CI)** |
| --- | --- |

**
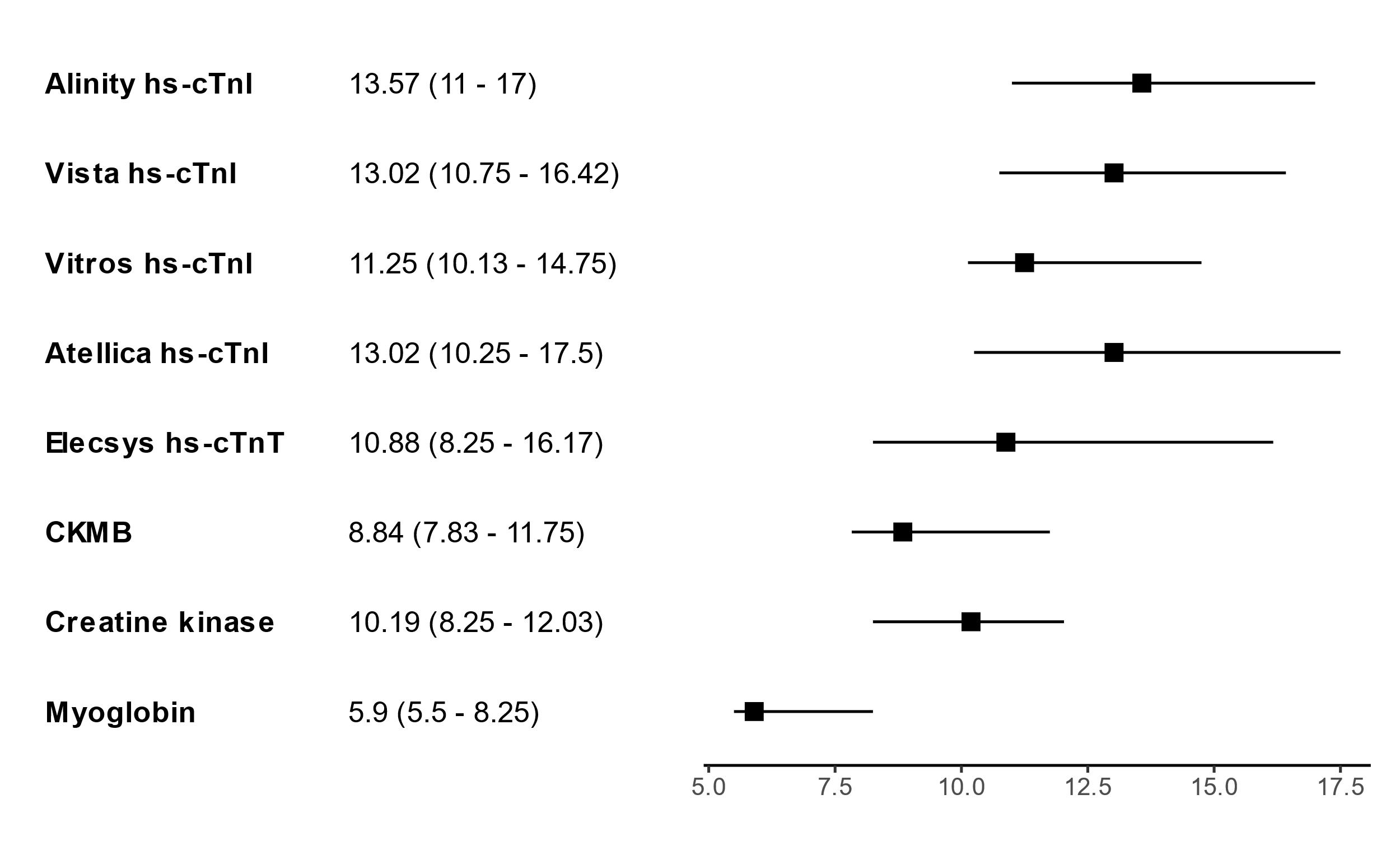
**

###
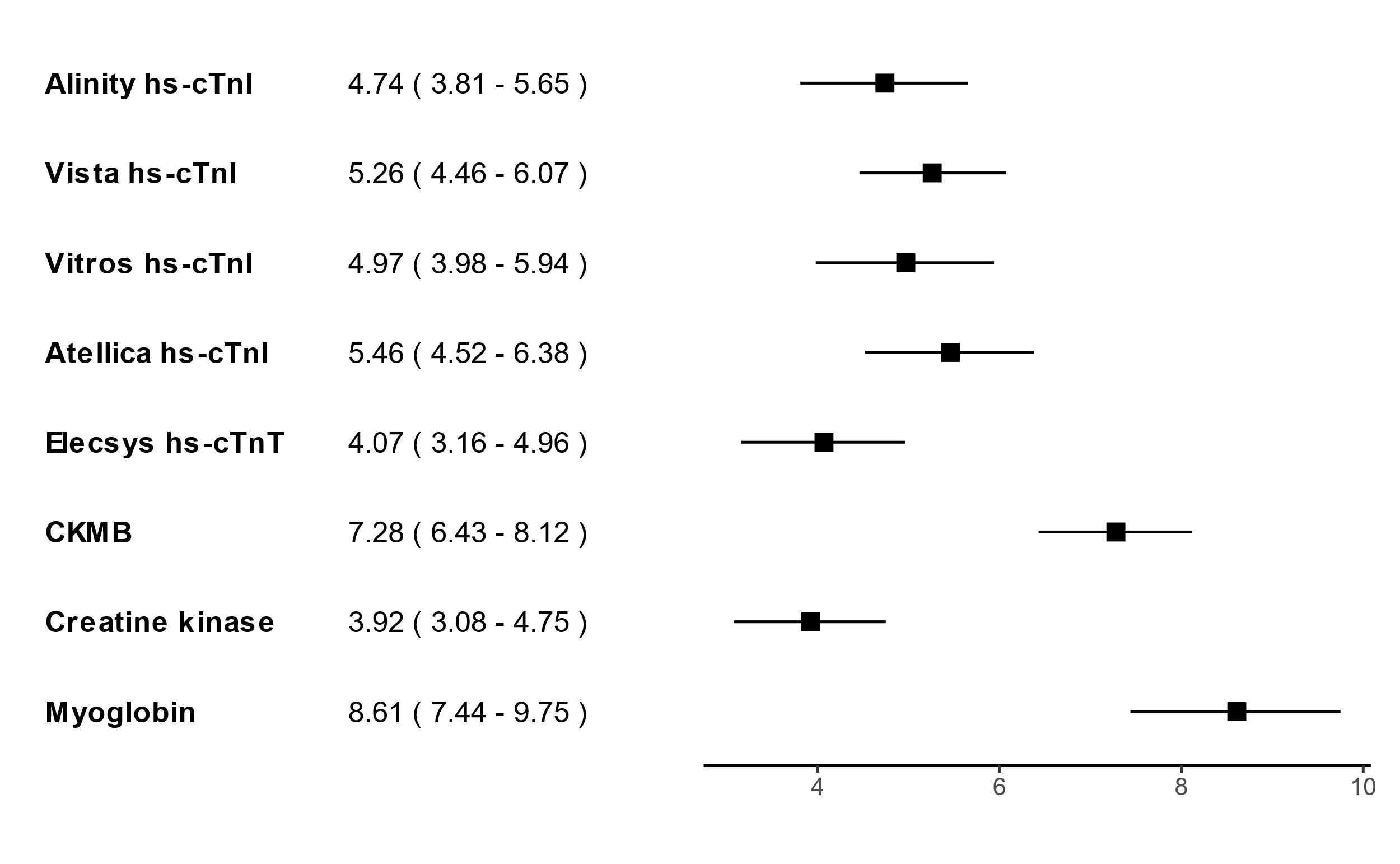
Panel C

| **Biomarker** | **Median rate of decay in percentage/hour (95%-CI)** |
| --- | --- |

###
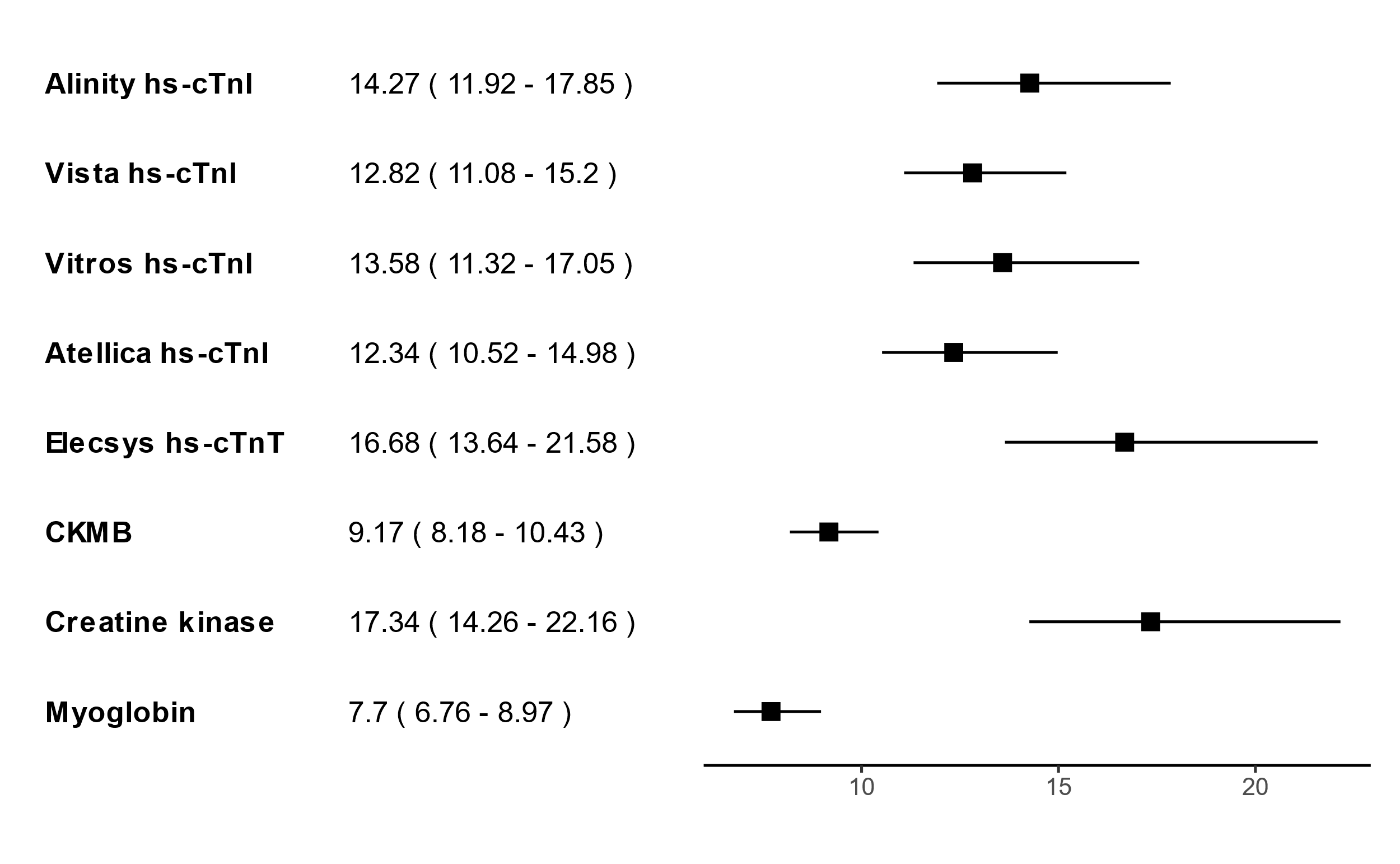
Panel D

| **Biomarker** | **Median half-life in hours (95%-CI)** |
| --- | --- |

Forrest plots of time to peak, rate of decay and half-life for high sensitivity (hs) cardiac troponins (cTn), creatine kinase (CK), CKMB, and myoglobin for participants with eGFR >90 ml/min/1,73m^2^.

Panel A: Median time in hours from PCI to peak concentration with 95% confidence intervals (CI).

Panel B: Median time in hours from percutaneous coronary intervention (PCI) to peak concentration.

Panel C: Median rate of decay from peak of concentration in hours with 95%-CI.

Panel D: Median half-life from peak of concentration in hours with 95%-CI.
